# Triosephosphate Isomerase and Its Product Glyceraldehyde-3-Phosphate Are Involved in the Regulatory Mechanism That Suppresses Exit from the Quiescent State in Yeast Cells

**DOI:** 10.1128/spectrum.00897-22

**Published:** 2022-08-04

**Authors:** Guoyu Liu, Yan Yang, Ganglong Yang, Shenglin Duan, Peng Yuan, Shuang Zhang, Feng Li, Xiao-Dong Gao, Hideki Nakanishi

**Affiliations:** a Key Laboratory of Carbohydrate Chemistry and Biotechnology, Ministry of Education, School of Biotechnology, Jiangnan University, Wuxi, China; b Beijing Key Laboratory of the Innovative Development of Functional Staple and Nutritional Intervention for Chronic Diseases, China National Research Institute of Food and Fermentation Industries Co., Ltd., Beijing, China; c The State Key Laboratory of Food Science and Technology, Jiangnan University, Wuxi, China; The Hebrew University-Hadassah School of Dental Medicine

**Keywords:** glyceraldehyde-3-phosphate, quiescent cells, spores, triosephosphate isomerase, yeasts

## Abstract

Cells of the budding yeast Saccharomyces cerevisiae form spores or stationary cells upon nutrient starvation. These quiescent cells are known to resume mitotic growth in response to nutrient signals, but the mechanism remains elusive. Here, we report that quiescent yeast cells are equipped with a negative regulatory mechanism which suppresses the commencement of mitotic growth. The regulatory process involves a glycolytic enzyme, triosephosphate isomerase (Tpi1), and its product, glyceraldehyde-3-phosphate (GAP). GAP serves as an inhibitory signaling molecule; indeed, the return to growth of spores or stationary cells is suppressed by the addition of GAP even in nutrient-rich growth media, though mitotic cells are not affected. Reciprocally, dormancy is abolished by heat treatment because of the heat sensitivity of Tpi1. For example, spores commence germination merely upon heat treatment, which indicates that the negative regulatory mechanism is actively required for spores to prevent premature germination. Stationary cells of Candida glabrata are also manipulated by heat and GAP, suggesting that the regulatory process is conserved in the pathogenic yeast.

**IMPORTANCE** Our results suggest that, in quiescent cells, nutrient signals do not merely provoke a positive regulatory process to commence mitotic growth. Exit from the quiescent state in yeast cells is regulated by balancing between the positive and negative signaling pathways. Identifying the negative regulatory pathway would provide new insight into the regulation of the transition from the quiescent to the mitotic state. Clinically, quiescent cells are problematic because they are resistant to environmental stresses and antibiotics. Given that the quiescent state is modulated by manipulation of the negative regulatory mechanism, understanding this process is important not only for its biological interest but also as a potential target for antifungal treatment.

## INTRODUCTION

In response to environmental stresses, microorganisms cease mitotic growth and form quiescent cells. Entering a quiescent state allows cells to maintain their viability in unfavorable conditions while retaining the ability to resume mitotic growth upon the resumption of favorable conditions (1). In the budding yeast Saccharomyces cerevisiae, one form of quiescence, termed the stationary phase, is induced by the depletion of various nutrients, including carbon and nitrogen sources and phosphorous (2, 3). Morphologically and physiologically, stationary-phase cells are distinct from mitotically growing cells; for example, transcription and translation levels are significantly decreased (4, 5), the cell wall is thicker than that in mitotic cells (6, 7), and the cells exhibit greater resistance to environmental stresses (8, 9). In yeast cells, exit from a stationary state is induced by the addition of glucose.

Some yeast cells, including S. cerevisiae, can form another type of quiescent cell termed spores, which are haploid cells produced via meiosis (10). Sporulation occurs when diploid cells are incubated in the presence of a nonfermentable carbon source and the absence of a nitrogen source. During sporulation, spores are created in the cytosol of the mother cell, where each nucleus produced via meiosis is enclosed in the newly synthesized spore plasma membrane and spore wall (11). Like the stationary phase, cellular activities are significantly decreased in spores; however, certain levels of transcription and translation activities are maintained (12). The morphology of spores is also distinct from that of mitotic cells. In particular, the spore wall is more complex than the cell wall in mitotic cells. The spore wall is composed of four layers, and the first outermost layer, the dityrosine layer, and the second outermost layer, the chitosan layer, are structures unique to the spore wall (11). These outer layers make spores resistant to environmental stresses (13–15). In response to a nutrient signal, spores resume mitotic growth. This process is termed germination (16). Glucose is known to be a strong inducer of germination, but the trigger for the process is most likely its intracellular metabolite(s) (17). Previous studies reported that the protein kinase A and target of rapamycin complex 1 (TORC1) signaling pathways are required to commence germination (17, 18), although the detailed mechanism to transmit the nutrient signal remains unknown.

Previously, we performed experiments in which spores released from asci were incubated with lysate of asci to analyze the assembly of the dityrosine layer (19). During the course of this study, we found that spores germinate when they are incubated with heat-treated lysate. This finding allowed us to identify inhibitors that suppress germination of spores, namely, triosephosphate isomerase (Tpi1) and its product, glyceraldehyde-3-phosphate (GAP). In the glycolysis pathway, cleavage of fructose-1,6-bisphosphate results in production of GAP and dihydroxyacetone phosphate (DHAP). Tpi1 is a homodimeric enzyme which mediates interconversion between GAP and DHAP (20). GAP is further metabolized to pyruvate in glycolysis. We demonstrate that exit from a quiescent state can be manipulated by modulation with Tpi1 or GAP. Thus, yeast cells are equipped with a negative regulatory mechanism to suppress their entrance into the mitotic state.

## RESULTS

### Spore germination is induced by heat treatment.

**FIG 1:**
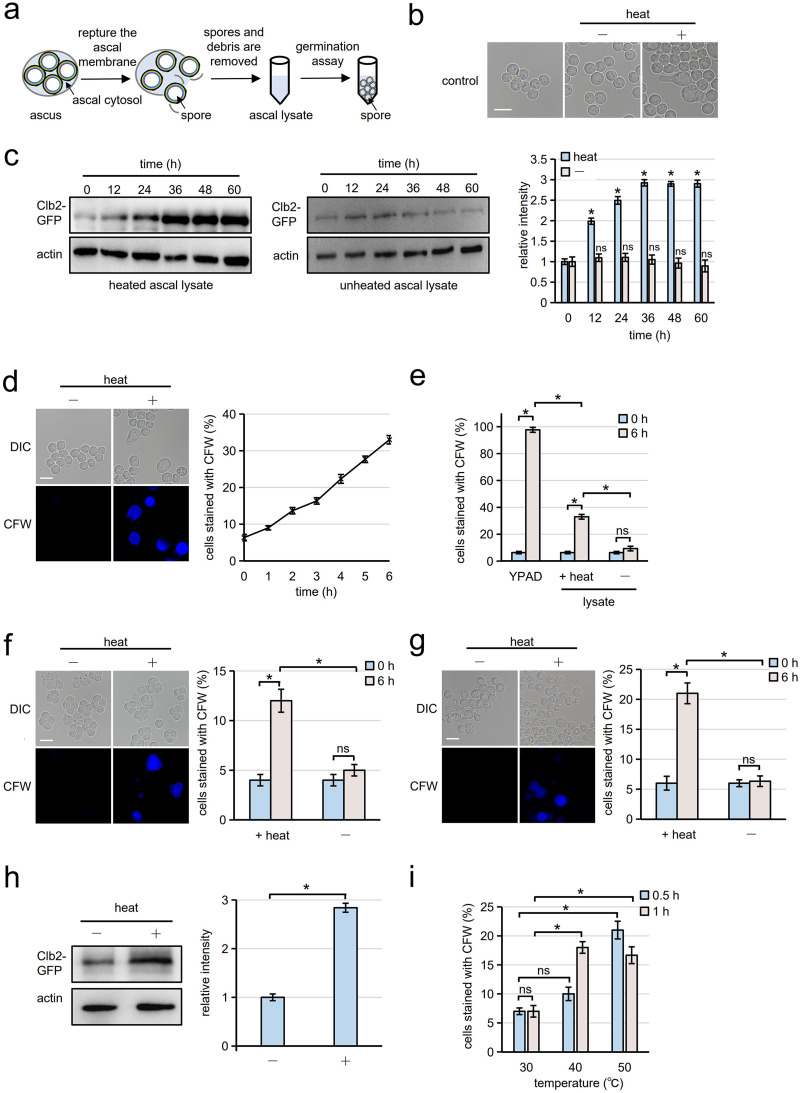
Germination of S. cerevisiae spores is induced by heat. (a) Schematic diagram for preparation of the ascal lysate and the germination assay using the ascal lysate. To prepare the ascal lysate, asci suspended in water were ruptured by sonication. After centrifugation, the supernatant, which contained the ascal cytosol (shown in blue), was incubated with spores to assay germination. (b) Spores were incubated with ascal lysate treated with (+) or without (−) heat (90°C, 10 min). The suspensions were incubated at 30°C for 6 h and observed by differential interference contrast (DIC) microscopy. Spores before incubation with ascal lysate are shown as controls (control). Scale bar, 5 μm. (c, Left panels) Spores were incubated in ascal lysate treated with or without heat (90°C, 10 min). The spore suspension was incubated at 30°C for indicated times, and Clb2-GFP was detected by Western blotting using an anti-GFP antibody. Actin was detected as loading control. (c, Right) Relative intensities of Clb2-GFP signals detected by Western blotting. The amount of Clb2-GFP detected in the lysate with 0 h of incubation was taken as 1. Student’s *t* test was performed between 0 h and each time point. (d) Spores were incubated with ascal lysate treated with (+) or without (−) heat (90°C, 10 min). The suspensions were incubated at 30°C for 6 h. After staining with CFW, DIC and fluorescence microscopy (CFW) images were obtained. Scale bar, 5 μm (left panels). The percentage of CFW-stained spores incubated in ascal lysate treated with heat was measured over time (right panel). Three hundred spores were analyzed for each assay. (e) Percentage of cells stained with CFW before (0 h) and after 6 h of incubation with heated (+heat) or unheated (−) lysate. Spores incubated with YPAD were also assayed. Three hundred spores were analyzed for each assay. (f, Left) Asci were treated with (+) or without (−) heat (at 95°C, 15 s) and incubated at 30°C for 6 h. After staining with CFW, DIC and fluorescence microscopy (CFW) images were obtained. Scale bar, 5 μm. (f, Right) Percentage of cells stained with CFW before (0 h) and after 6 h of incubation. Three hundred asci were analyzed for each assay. (g, Left) Spores released from asci were treated with (+) or without (−) heat (95°C, 15 s) and incubated at 30°C for 6 h. After staining with CFW, DIC and fluorescence microscopy (CFW) images were obtained. Scale bar, 5 μm. (g, Right) Percentage of cells stained with CFW before (0 h) and after 6 h of incubation. Three hundred spores were analyzed for each assay. (h, Left) Spores expressing Clb2-GFP were treated with heat (95°C, 15 s) and incubated at 30°C for 36 h. Clb2-GFP was detected in the lysate by Western blotting using an anti-GFP antibody. Actin was detected as a loading control. (h, Right) Relative intensities of Clb2-GFP signals detected by Western blotting. The level of Clb2-GFP detected in the lysate with 0 h of incubation was taken as 1. (i) Spores were incubated at the indicated temperatures for 0.5 or 1 h. The spores were incubated at 30°C for 6 h, and germination efficiency was assayed with CFW staining. Three hundred spores were analyzed for each assay. The data in panels c to i are presented as the mean ± SE; *n* = 3. Statistical analysis was performed by two-tailed unpaired Student’s *t* tests; *, *P < *0.05; ns, not significant.

S. cerevisiae spores are enclosed in asci. In this study, extraspore fluid in the ascus is referred to as ascal cytosol (Fig. 1a). Asci can be ruptured by sonication without breaking the spores. After centrifugation of sonicated asci, the supernatant is referred to as ascal lysate (Fig. 1a). We found that the morphology of spores was altered when spores were incubated with ascal lysate heat treated at 90°C for 10 min (Fig. 1b). No morphological change was observed when the spores were incubated in unheated ascal lysate. We speculated that the morphological change was attributable to germination. To verify this hypothesis, the mitotic cyclin Clb2 was expressed as a green fluorescent protein (GFP) fusion protein (Clb2-GFP) from its own promoter, and its expression levels were assessed. As reported before (21), expression of Clb2 was upregulated in germinating cells (see Fig. S1 in the supplemental material). We found that expression levels of Clb2-GFP were increased by incubation with heat-treated ascal lysate (Fig. 1c). To analyze further the mechanism of heat-induced germination, we sought a convenient method to detect germinating cells so that we could quantify germination efficiency. Spores have the chitosan layer in the spore wall (22), but they are not stained with the chitosan binding dye, calcofluor white (CFW), because the outermost layer prevents binding of the dye to the chitosan layer (Fig. S2a) (23). However, we found that spores were stained with CFW when they were incubated in nutrient-rich medium (YPAD medium) supplemented with 2% glucose (Fig. S2a), presumably because the spore wall became CFW permeable during germination. Dead spores were not stained with CFW after incubation in YPAD medium (Fig. S2a). When spores were incubated in YPAD medium, the number of CFW staining-positive spores was increased, which correlated with an increase in the number of swollen and deformed cells (Fig. S2b). These results showed that CFW staining is a useful assay to quantify the germination efficiency of spores. As shown in Fig. 1d, spores stained with CFW were increased when spores were incubated in heat-treated ascal lysate (Fig. 1d), although the progress of germination of heat-treated spores was slow compared to that of YPAD medium-induced germination (Fig. 1e). This increase in CFW staining was not seen when spores were incubated with unheated lysate (Fig. 1e).

We then examined whether spores germinate when they are directly treated with heat. Spores in asci germinated when they were incubated at 95°C for 15 s (Fig. 1f). Heat-induced germination was also observed for spores released from asci (here referred to simply as spores); thus, ascal cytosol is not necessary to induce germination (Fig. 1g and Fig. S3). We verified that Clb2-GFP levels were increased by heat treatment (Fig. 1h). It should be noted that Clb2-GFP levels in heat-treated spores were analyzed after 36 h incubation at 30°C because, as shown in Fig. 1c, Clb2-GFP reached a plateau after 36 h when spores were incubated in heat-treated ascal lysate. Germination efficiencies were assessed at various temperatures and treatment times (Fig. 1i and Fig. S3). This assay was performed after a subsequent 6-h incubation at 30°C. Germination was induced by 70°C or higher heat treatments for 15 s (Fig. S3). However, germination can be induced at lower temperatures when time of heat treatment is extended; we found that germination could be induced when spores were incubated at 40°C for 1 h (Fig. 1i).

### Ascal lysate contains an inhibitor to suppress germination.

**FIG 2:**
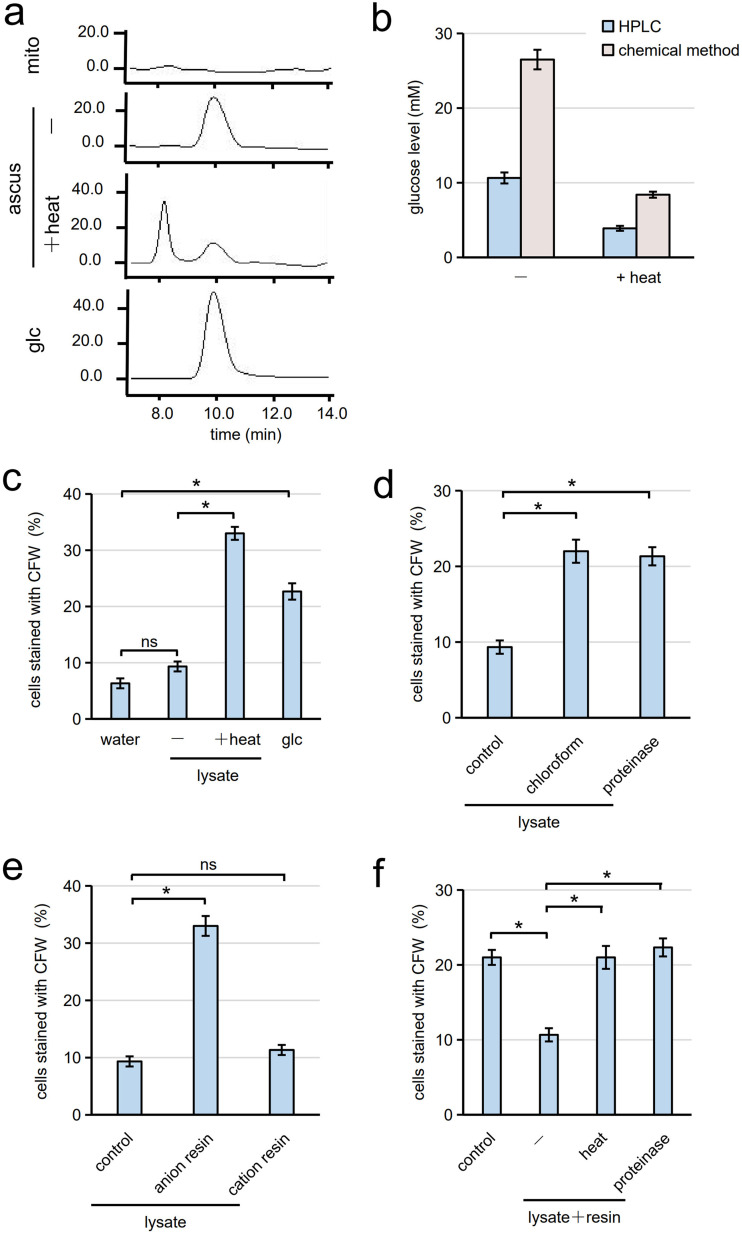
Ascal lysate contains an inhibitor to suppress germination. (a) Detection of glucose in mitotic cell lysate (mito) and ascal lysate (ascus) before (−) or after (+heat) heat treatment (90°C, 10 min) by HPLC. Glucose solution (glc) was used as a standard. (b) The glucose concentration in ascal lysate before (−) and after (+heat) heat treatment was measured by HPLC or chemical methods. (c) Spores suspended in water, 0.2% glucose solution (glc), or ascal lysate (lysate) treated with (+heat) or without (−) heat (90°C, 10 min) were incubated at 30°C for 6 h. After staining with CFW, cells stained with CFW were counted under the microscope. Three hundred spores were analyzed for each assay. (d) Spores were suspended in ascal lysate treated with or without (control) chloroform extraction (chloroform) or proteinase. These spore suspensions were incubated at 30°C for 6 h, and germination efficiency was assayed with CFW staining. Three hundred spores were analyzed for each assay. (e) Ascal lysate incubated with or without (control) anion or cation exchange resin was centrifuged, and the supernatant was then incubated with spores. After incubation at 30°C for 6 h, germination efficiency was assayed with CFW staining. Three hundred spores were analyzed for each assay. (f) Anion exchange resin was incubated with ascal lysate (lysate). The resin was collected and treated with or without (−) heat (95°C, 15 s) or proteinase. The resins and spores were suspended in 0.2% glucose solution and incubated at 30°C for 6 h. Germination efficiency was assayed with CFW staining. As a control, spores were incubated with anion exchange resin in 0.2% glucose solution. Three hundred spores were analyzed for each assay. The data in panels b to f are presented as the mean ± SE; *n* = 3. Statistical analysis was performed by two-tailed unpaired Student’s *t* tests. *, *P < *0.05; ns, not significant.

We were interested in the mechanism underlying the induction of heat-induced germination. We first hypothesized that a germination inducer is produced by heat treatment. Germination is known to be induced by incubation with glucose. Thus, we analyzed the glucose levels in ascal lysate before and after heat treatment. High-performance liquid chromatography (HPLC) analysis of glucose levels showed that the levels were not increased by heat treatment either at 90°C for 10 min or at 95°C for 15 s (Fig. 2a and Fig. S4). However, it is notable that glucose was present in ascal lysate at a concentration of 11 mM (0.2%) (Fig. 2a and Fig. S4). As a control, lysate was prepared from mitotic cells cultured in YPAD medium in a manner similar to ascal lysate. No glucose was detected in the lysate (Fig. 2a), presumably because the glucose concentration was below the detection limit. The presence of glucose in ascal lysate was further verified by measuring glucose levels with a chemical method; glucose concentrations in ascal lysate before and after heat treatment (90°C for 10 min) were 26 mM and 8 mM, respectively (Fig. 2b). When spores were incubated with 0.2% glucose solution, germination was induced (Fig. 2c). These results led us to propose another hypothesis: an inhibitor which can suppress germination is present in ascal cytosol. In this hypothesis, heat-induced germination is attributable to inactivation of the inhibitor. In line with this hypothesis, we found that germination was induced with ascal lysate after treatment by chloroform extraction (Fig. 2d). Germination was also induced by ascal lysate treated with proteinase (Fig. 2d), indicating that the inhibitor is a protein. In addition, we found that the inhibitor was adsorbed by anion exchange resin. Indeed, germination was induced when spores were incubated with the supernatant of ascal lysate mixed with anion exchange resin, but not with cation exchange resin (Fig. 2e). Anion exchange resin adsorbed with the inhibitor could inhibit the germination of spores in 0.2% glucose solution (Fig. 2f). Like ascal lysate, the inhibitor bound to the resin was inactivated by heat or proteinase treatment (Fig. 2f).

### Tpi1 present in ascal lysate suppresses germination.

**FIG 3:**
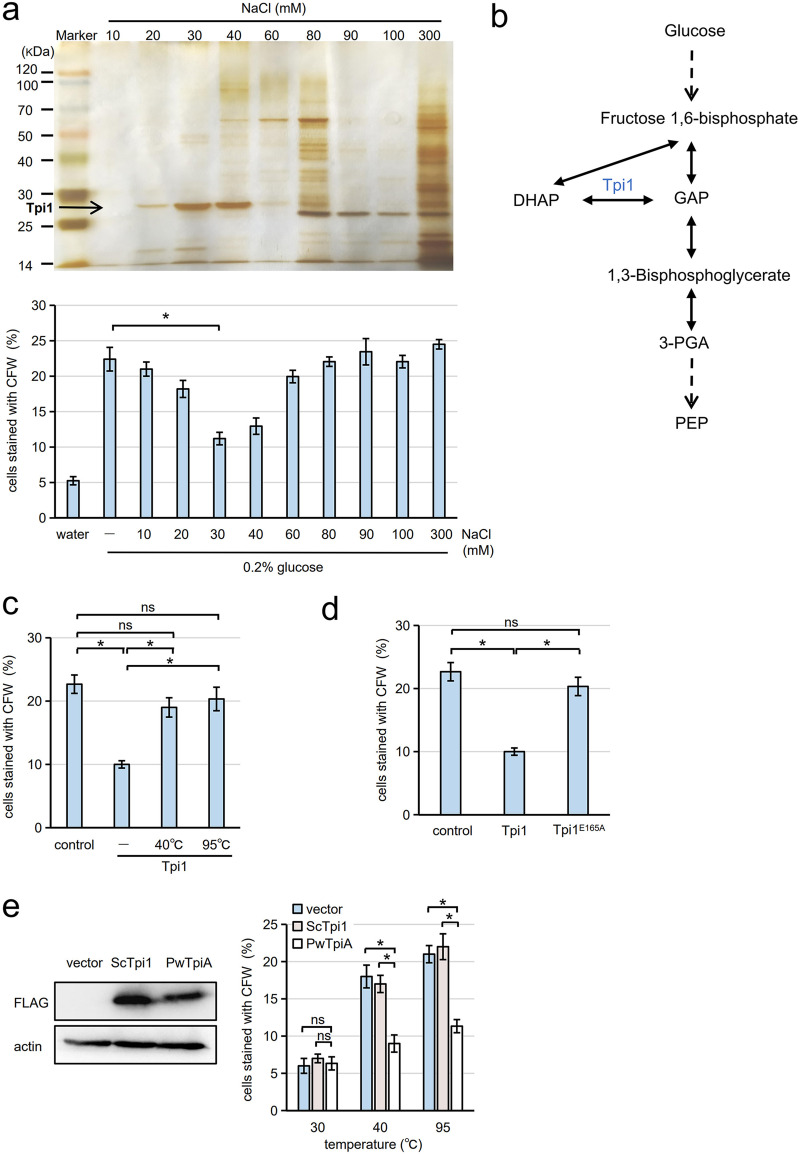
Tpi1 is the inhibitor present in ascal lysate. (a, Top) Ascal lysate treated as shown in Fig. S5a in the supplemental material was applied to anion exchange resin column. Proteins eluted with indicated concentration of NaCl solutions were separated by SDS-PAGE and detected with silver staining. The arrow indicates the Tpi1 band. (a, Bottom) Spores were incubated with the eluents in the presence of 0.2% glucose, and germination efficiency was assayed with CFW staining. As controls, germination efficiencies of spores incubated with water or 0.2% glucose solution (−) are shown. Three hundred spores were analyzed for each assay. (b) Schematic of glycolytic pathway from glucose to phosphoenolpyruvate (PEP). (c) Spores were incubated in 0.2% glucose solution supplemented with or without (control) recombinant Tpi1. Tpi1 was treated with or without (−) heat (40°C for 1 h or 95°C for 15 s). The spore suspensions were incubated at 30°C for 6 h. After staining with CFW, cells stained with CFW were counted under the microscope. Three hundred spores were analyzed for each assay. (d) Spores were incubated in 0.2% glucose solution with or without (control) wild-type Tpi1 or Tpi1^E165A^. The spore suspensions were incubation at 30°C for 6 h, and germination efficiency was assayed with CFW staining. Three hundred spores were analyzed for each assay. (e, Left panels) S. cerevisiae *TPI1-FLAG* (ScTpi1) or P. woesei *tpiA-FLAG* (PwTpiA) expressed in yeast cells was detected by Western blotting (FLAG). Yeast cells harboring empty vector (vector) were used as a control. Actin was detected as a loading control. (e, Right) After cells harboring the plasmids sporulated, the spores released from asci were heated at 30°C for 1 h, 40°C for 1 h, or 95°C for 15 s. Then, the spores were incubated at 30°C for 6 h, and germination efficiency was assayed with CFW staining. Three hundred spores were analyzed for each assay. The data in panels a and c through e are presented as the mean ± SE; *n* = 3. Statistical analysis was performed by two-tailed unpaired Student’s *t* tests. *, *P < *0.05; ns, not significant.

To identify the proteinaceous inhibitor present in ascal lysate, the inhibitor was purified using anion exchange resin. The purification procedure is briefly described in Fig. S5a. When proteins bound to the anion exchange resin column were eluted with serial concentrations of salt solution, we found that the fraction eluted with 30 mM NaCl exhibited the highest activity to inhibit germination (Fig. 3a). The primary protein band in this fraction was detected between 25 and 30 kDa by SDS-PAGE (Fig. 3a). The protein band was excised from the gel and subjected to high-resolution mass spectrometry (MS) analysis. The analysis showed that Tpi1 (26.8 kDa) was the primary protein included in the band (10 unique peptides and 46% coverage of the proteins) (Table S1 and Fig. S5b). All peptides were confirmed by MS/MS spectra manually. Other proteins were identified with only one or two peptides (Table S1).

Tpi1 is a glycolytic enzyme that catalyzes the interconversion between DHAP and GAP (Fig. 3b). To confirm whether Tpi1 could suppress spore germination, a germination assay was performed in the presence of recombinant Tpi1 produced by Escherichia coli. The recombinant enzyme retains catalytic activity and could produce GAP from DHAP *in vitro* (Fig. S6). As shown in Fig. 3c, the germination of spores in 0.2% glucose solution was suppressed by the addition of recombinant Tpi1. Like the inhibitor found in ascal lysate, the inhibitory activity of recombinant Tpi1 was abolished by heat treatment (40°C for 1 h or 95°C for 15 s) (Fig. 3c). We verified that the recombinant Tpi1 was catalytically inactivated by incubation at 40°C for 1 h or 95°C for 15 s (Fig. S6). To further assess whether catalytic activity of Tpi1 is required to suppress germination, a catalytically inactive Tpi1 mutant, Tpi1^E165A^ (24), was produced. Recombinant Tpi1^E165A^ did not suppress the germination of spores in 0.2% glucose solution (Fig. 3d). These results suggest that the enzymatic product of Tpi1 would be a downstream component required for the inhibitory process.

To analyze the effect of the loss of Tpi1 in germination, we constructed diploid *tpi1*Δ cells. As reported before (25), *tpi1*Δ cells were viable when ethanol was supplemented (Fig. S7a). However, the mutant cells were unable to form spores (Fig. S7b). Thus, to verify that heat-induced germination is triggered by inactivation of Tpi1 in spores, we prepared spores harboring a thermotolerant triosephosphate isomerase. If heat-induced germination is attributable to the inactivation of Tpi1, germination would be suppressed by the expression of a thermotolerant triosephosphate isomerase. Pyrococcus woesei is a thermophilic bacteria, and a previous study showed that its triosephosphate isomerase, TpiA, is a thermotolerant enzyme (half-life at 104°C is 280 min) (26). Spores released from asci were treated with heat, and gemination efficiency was assayed. Compared to control spores in which yeast *TPI1* was similarly expressed from a plasmid, the levels of heat-induced germination were significantly decreased in spores expressing P. woesei *tpiA* (Fig. 3e). Thus, Tpi1 serves as an inhibitor both in ascal cytosol and in spores.

### GAP inhibits spore germination.

**FIG 4:**
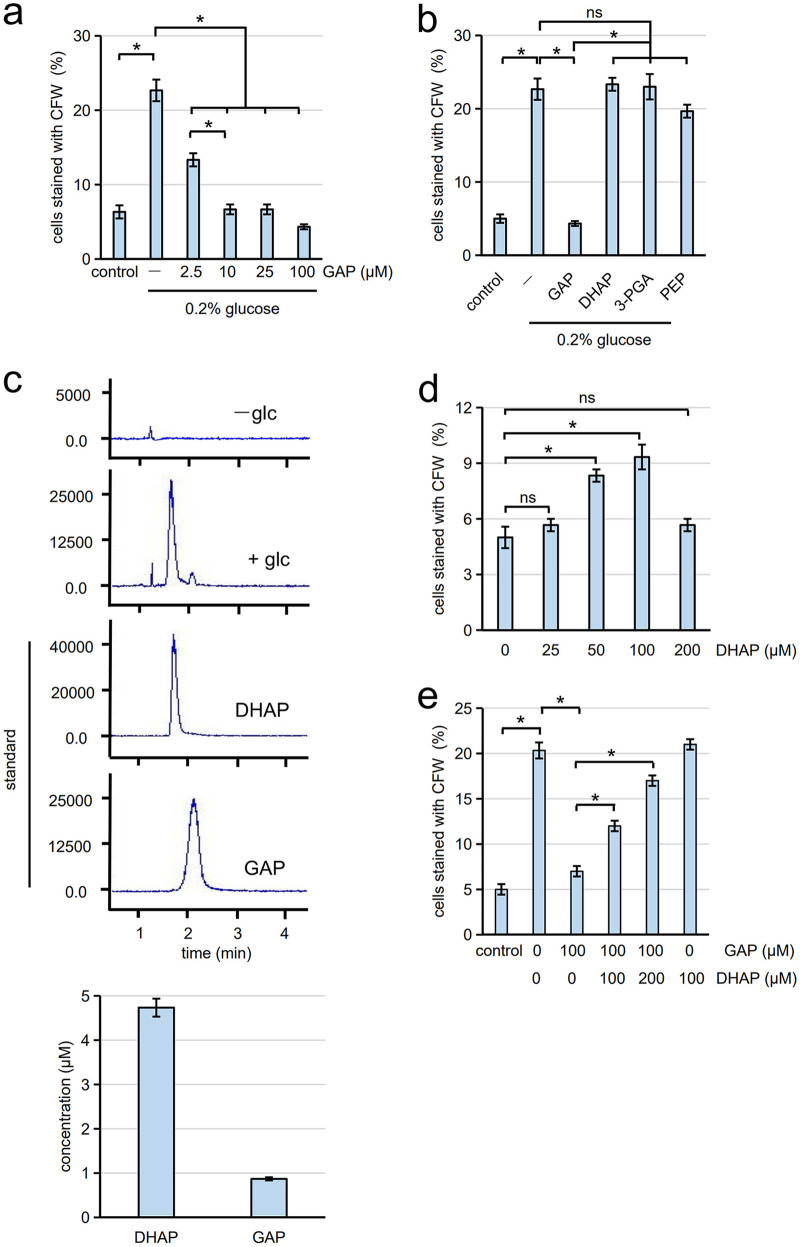
GAP can inhibit germination. (a) Spores were incubated with water (control) or 0.2% glucose supplemented with the indicated concentrations of GAP. After incubation at 30°C for 6 h, germination efficiency was measured with CFW staining. Three hundred spores were analyzed for each assay. (b) Spores were incubated with water (control) or 0.2% glucose supplemented with 100 μM GAP, DHAP, 3-PGA, or PEP. After incubation at 30°C for 6 h, germination efficiency was assayed with CFW staining. Three hundred spores were analyzed for each assay. (c, Top panels) Spores were incubated with 0.2% glucose solution (+glc) or water (−glc) at 30°C for 1 h. The supernatant of the spore suspensions was subjected to LC-MS to detect DHAP, and liquid chromatography data are shown. Mass spectrometry data are shown in Fig. S6 in the supplemental material. DHAP and GAP were subjected to LC-MS as standard samples. (c, Bottom panels) Quantification of DHAP and GAP detected in the assay solution. (d) Spores were suspended in water supplemented with the indicated amount of DHAP and incubated at 30°C for 6 h. Germination efficiency was assayed with CFW staining. Three hundred spores were analyzed for each assay. (e) Spores treated with or without (control) heat (40°C, 1 h) were suspended in water supplemented with indicated amounts of GAP and DHAP. After incubation at 30°C for 6 h, germination efficiency was assayed with CFW staining. Three hundred spores were analyzed for each assay. The data are presented as the mean ± SE; *n* = 3. Statistical analysis was performed by two-tailed unpaired Student’s *t* tests. *, *P < *0.05; ns, not significant.

The enzymatic products of Tpi1 are DHAP or GAP. In glycolysis, GAP is the primary product of Tpi1. We found that the germination of spores in 0.2% glucose was significantly inhibited in the presence of 10 μM GAP (Fig. 4a). DHAP did not inhibit germination, even at 100 μM (Fig. 4b). We also examined two other glycolysis products, 3-phosphoglycerate (3-PGA) and phosphoenolpyruvate (PEP), which are downstream processing products of GAP (Fig. 3b), but neither suppressed germination (Fig. 4b). Thus, GAP serves as an inhibitory molecule for germination.

The results described above suggest that Tpi1 present outside spores produces GAP when spores are incubated with glucose. However, it is unlikely that glucose is metabolized outside spores. Thus, we speculated that DHAP was generated by the spores. To test this hypothesis, spores were incubated with glucose, and the supernatant was subjected to liquid chromatography-mass spectrometry (LC-MS) analysis to detect DHAP. Although DHAP and GAP exhibit the same fragmentation pattern in MS analysis (Fig. S8a), they can be separated by HPLC as shown in Fig. S6. Strikingly, we detected DHAP in the supernatant when spores were incubated in 0.2% glucose for 1 h (Fig. 4c and Fig. S8b). In this condition, 4.7 μM DHAP and 0.9 μM GAP were detected in the assay solution (Fig. 4c). These results show that asci are equipped with a unique mechanism to generate GAP in the ascal cytosol.

Given that GAP serves as an inhibitor for germination, heat-induced germination may be triggered by decreased levels of GAP. However, apart from GAP reduction, inactivation of Tpi1 also leads to an increase in DHAP levels. Thus, DHAP may be involved in triggering germination. Notably, we found that germination was slightly (approximately 5%) induced when spores were incubated with 100 μM DHAP, although this effect was not observed at a higher concentration (200 μM) of DHAP for unknown reasons (Fig. 4d). Thus, we examined whether DHAP could release the inhibitory effect of GAP. For this analysis, we used heat-treated spores to prevent conversion of DHAP to GAP. The germination of heat-treated spores can be inhibited by the addition of GAP (Fig. 4e). However, the inhibitory effect of GAP was alleviated by the addition of DHAP (Fig. 4e), suggesting that heat-induced germination may not merely be attributable to a decrease in GAP levels and that DHAP may be involved in the regulation of germination.

### The germination inhibitor (negative regulatory process) suppresses reentry of stationary cells into the cell cycle.

**FIG 5:**
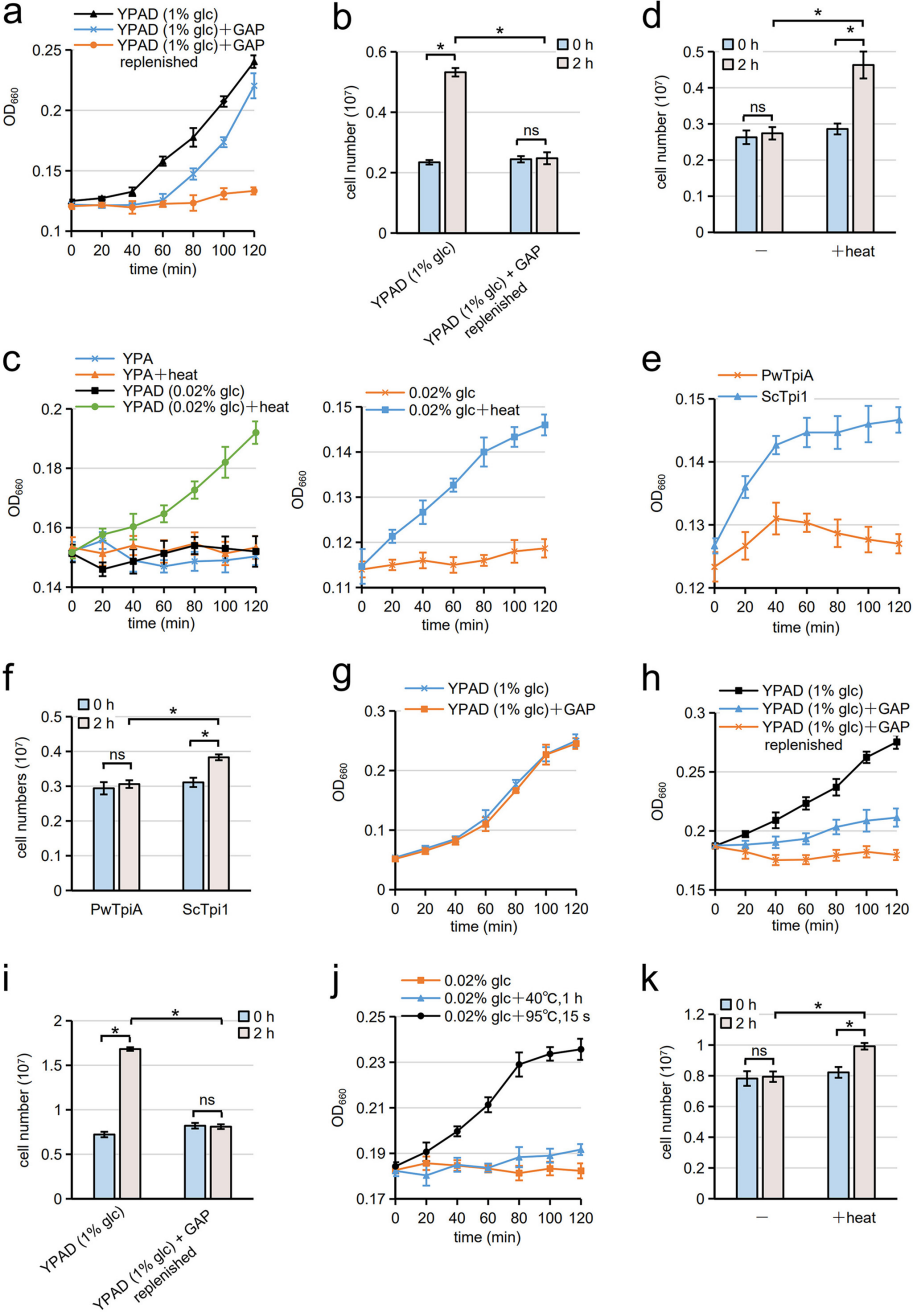
Exit from stationary state is suppressed by the negative regulatory process. (a and b) Stationary S. cerevisiae cells (haploid cells) were incubated in YPAD (1% glucose) supplemented with or without GAP at 30°C. The initial concentration of GAP in the medium was 50 μM. GAP was either replenished (0.1 μmol of GAP was added every 20 min) or not. (a) Cell growth was assayed by measuring turbidity. (b) Cell number was counted with a platelet counter before (0 h) and after (2 h) the incubation in YPAD or YPAD replenished with GAP every 20 min. (c) Stationary S. cerevisiae cells (haploid cells) treated with (heat) or without heat (40°C, 1 h) were incubated in YPA (glucose free) or YPAD (0.02% glucose) (left) or in water supplemented with 0.02% glucose (right). Cells were incubated at 30°C, and their growth was assayed by measuring turbidity. (d) Stationary S. cerevisiae cells (haploid cells) treated with (+heat) or without (−) heat (40°C, 1 h) were incubated in water supplemented with 0.02% glucose at 30°C for 2 h. Cell number was counted with a platelet counter before (0 h) and after (2 h) the incubation. (e and f) Stationary S. cerevisiae cells harboring *TPI1-FLAG* (ScTpi1) or P. woesei *tpiA-FLAG* (PwTpiA) (diploid cells used in Fig. 3e) were treated with (+heat) or without heat (40°C, 1 h). (e) The cells were incubated in water supplemented with 0.02% glucose at 30°C, and their growth was assayed by measuring turbidity. (f) Cell number was counted with a platelet counter before (0 h) and after (2 h) the incubation. (g) S. cerevisiae cells (haploid cells) in the logarithmic growth phase were incubated in YPAD (1% glucose) supplemented with or without GAP at 30°C, and their growth was assayed by measuring turbidity. We replenished 0.1 μmol of GAP every 20 min. The initial concentration of GAP in the medium was 50 μM. (h and i) Stationary C. glabrata cells were incubated in YPAD (1% glucose) supplemented with or without GAP at 30°C. The initial concentration of GAP was 50 μM. GAP was either replenished (0.1 μmol of GAP was added every 20 min) or not. (h) Cell growth was assayed by measuring turbidity. (i) Cell number was counted with a platelet counter before (0 h) and after (2 h) the incubation in YPAD or YPAD replenished with GAP every 20 min. (j and k) Stationary C. glabrata cells treated with (+heat) or without (−) heat (40°C for 1 h or 95°C for 15 s) were incubated in water supplemented with 0.02% glucose. (j) Cells were incubated at 30°C, and their growth was assayed by measuring turbidity. (k) Cell number was counted with a platelet counter before (0 h) and after (2 h) the incubation for the cells treated with (+ heat) or without (−) heat (95°C, 15 s). The data are presented as the mean ± SE; *n* = 3. Statistical analysis was performed by two-tailed unpaired Student’s *t* tests. *, *P < *0.05; ns, not significant.

In spores, we found that the transition from quiescent to mitotic growth is suppressed via a negative regulatory process. Thus, we assessed whether the regulatory process functions in other developmental states. Stationary cells can resume proliferation in response to nutrients; for example, when stationary cells were incubated in YPAD supplemented with 1% glucose, they started to proliferate (Fig. 5a). However, in the presence of 50 μM GAP, proliferation of the cells was delayed by approximately 1 h (Fig. 5a). We speculated that the growth of stationary cells was inhibited by GAP, but they could start proliferating when the GAP was consumed. Indeed, the growth of stationary cells was abolished when 0.1 μmol GAP was added every 20 min (initial concentration of GAP in the media was 50 μM). Cell growth was analyzed by turbidity (Fig. 5a) or counting the number of cells (Fig. 5b). Next, we examined whether stationary cells resume proliferation upon heat treatment. First, stationary cells treated with heat (40°C, 1 h) were suspended in glucose-free YPAD (YPA) medium. The cells could not proliferate under this condition (Fig. 5c), presumably because the carbon source was insufficient for proliferation. Thus, heat-treated stationary cells were incubated in YPAD medium with a glucose concentration of 0.02%. In this condition, stationary cells did not proliferate (Fig. 5c), but remarkably, we found that heat-treated cells grew under this condition (Fig. 5c). Heat-treated stationary cells also grew in water supplemented with 0.02% glucose (Fig. 5c and d). In stationary cells harboring P. woesei TpiA, growth was not induced by heat treatment (Fig. 5e and f), indicating that exit from stationary state can be induced by inactivation of Tpi1. Unlike stationary cells, the growth of mitotic cells (cells in the logarithmic growth phase) was not compromised by the addition of GAP (Fig. 5g). Thus, the negative regulatory process suppresses exit from quiescence but not mitotic divisions. We examined whether stationary *tpi1Δ* cells resume proliferation in YPAD medium containing 0.02% glucose, or 0.03% ethanol and 0.02% glucose. However, the mutant cells did not grow in the media even after treatment with heat (40°C, 1 h); levels of turbidity were not increased after 12 h incubation (Fig. S9). Since *tpi1*Δ cells exhibit growth defect (Fig. S7c), the mutant cells may not be able to grow in the media with limited carbon sources.

Next, we examined whether the negative regulatory mechanism is present in the pathogenic yeast Candida glabrata. Since C. glabrata cells do not undergo a sexual cycle (27), the effect of GAP was assessed in stationary cells. Like S. cerevisiae, the proliferation of C. glabrata cells in the stationary state was inhibited by the addition of GAP (Fig. 5h and i). Growth of C. glabrata stationary cells in water supplemented with 0.02% glucose was slightly induced by incubation at 40°C for 1 h (Fig. 5j). Their growth was improved when heat treatment was performed at 95°C for 15 s (Fig. 5j and k). Notably, proliferation of stationary-phase C. glabrata cells was not induced by heat treatment when they were incubated in YPAD medium containing 0.02% glucose (Fig. S10). Nevertheless, these results suggest that the negative regulatory mechanism is conserved among yeast species.

## DISCUSSION

We found that heat alone triggers spore germination as assayed by chitin exposure or Clb2 level. Since germination is known to be induced by glucose, it was somewhat surprising that spores commence germination merely by heat treatment. Analysis of heat-induced germination revealed the presence of a negative regulatory process which maintains dormancy in yeast cells. GAP and Tpi1 are negatively regulating exit from quiescence (a G_0_-to-G_1_ transition) but not progression through the cell cycle. This is consistent with mitotic cells being unaffected by GAP.

Sporulation is induced when the environment is nutrient poor. However, spores are formed in the cytosols of the mother cells (11), which likely contain certain levels of nutrient. We detected glucose in ascal lysate, and its concentration was more than 10 mM. The lysate was prepared by rupturing asci suspended in water, and thus, the glucose levels in ascal cytosol may actually be higher. Our results suggest that the glucose levels in mitotic cells are significantly lower than those in ascal cytosol. A previous study showed that the glucose concentration in mitotic cells was 228 μM when cells were incubated in 0.5% glucose and 100 nM or lower when incubated in 2% ethanol (28). The relatively high levels of glucose found in ascal lysate in the present study might be due to glucose levels in ascal cytosol being increased during spore formation; for example, perhaps glucose is released from the spore wall when the glucan layer is produced. Alternatively, we cannot rule out the possibility that glucose levels could be artificially increased during preparation of the lysate. Nevertheless, here, we demonstrate that ascal lysate does not induce germination because of the presence of an inhibitor. Spores within asci commence germination when this regulatory mechanism is abolished by heat treatment. Thus, we propose that the regulatory process is required to prevent spores from germinating prematurely.

**FIG 6:**
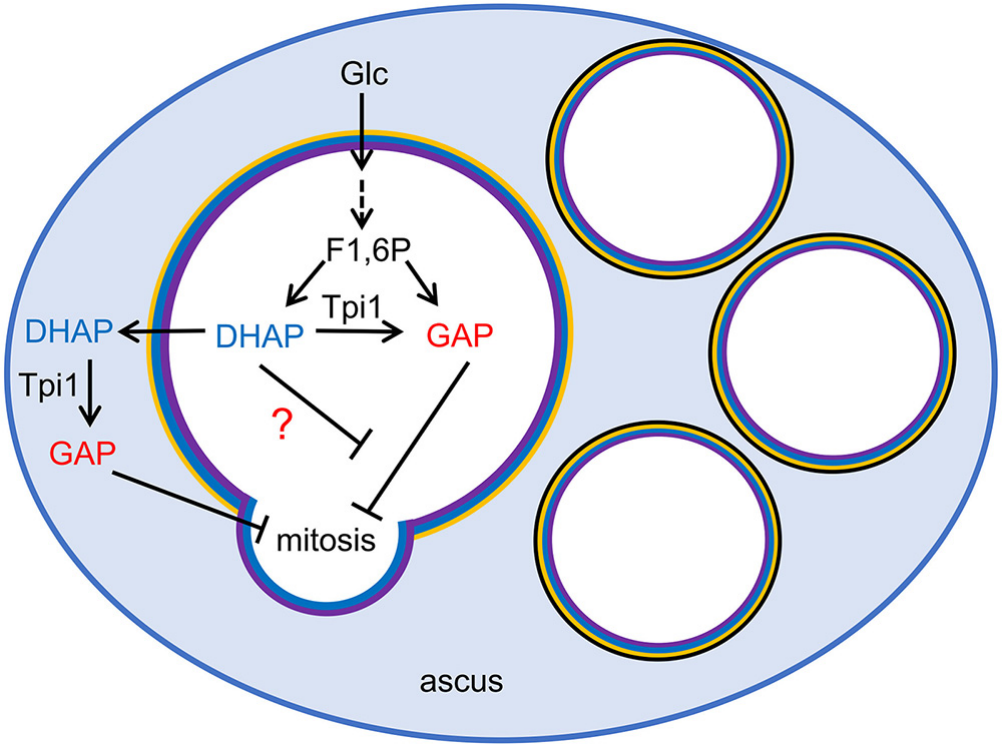
Model of the mechanism to suppress germination in the ascus. Tpi1 and GAP are involved in the negative regulatory process to suppress reentry into mitosis. In asci, GAP is also produced in the ascal cytosol by Tpi1 because Tpi1 is present in the ascal cytosol, and DHAP is secreted from spores. GAP generated in ascal cytosol is internalized in spores and inhibits germination.

Unlike wild-type cells, stationary *tpi1Δ* cells do not grow in YPAD medium supplemented with 0.02% glucose and 0.03% ethanol regardless of heat treatment. We could not obtain decisive results from the experiments using *tpi1Δ* cells; thus, we cannot rule out the possibility that inactivation of Tpi1 may not be the sole trigger for heat-induced exit from the quiescent state. For example, modulation of other metabolic enzyme(s) may contribute to abrogating the negative regulatory pathway. Nevertheless, our results suggest that Tpi1 is involved in the negative regulatory pathway, and its product, GAP, serves as an inhibitory molecule, although the downstream targets of GAP are currently unknown. GAP must be metabolized in yeast cells via the glycolysis pathway or pentose phosphate pathway. Thus, GAP should be continuously produced to suppress commencement of mitotic growth in the presence of glucose. The negative regulatory mechanism most likely raises the threshold for the glucose level required to reenter mitotic growth in spores and stationary cells. GAP is regulating cell cycle reentry rather than progression. Notably, asci are equipped with a unique mechanism in which GAP is produced via Tpi1 present in the ascal cytosol (Fig. 6). In this process, the substrate DHAP is secreted from the spores, indicating that spores have transporters for DHAP and GAP in the spore plasma membrane. We demonstrated that higher levels of GAP make spores more impervious to the germination signal, and so, in asci, the negative regulatory mechanism is augmented by GAP produced in ascal cytosol. Our results suggest that the mechanism is required for spores to maintain the quiescent state in asci because the ascal cytosol contains a high concentration of glucose.

In spores, glucose and/or its metabolites should serve as signaling molecules to induce germination. However, glucose may not simply activate the signaling pathway to initiate germination because GAP is a glycolytic metabolite. Our results suggest that the negative regulatory process functions when glucose concentrations are low, but the positive regulating pathway should override the negative regulation by certain levels of glucose. Thus, switching between negative and positive regulatory processes may regulate the initiation of germination. Given that GAP serves as an inhibitory molecule, the transition between the quiescent and mitotic state may be regulated by GAP levels. The fact that germination is induced by inactivation of Tpi1 appears consistent with this hypothesis. However, in the glycolysis pathway, production of GAP is not abrogated by the loss of Tpi1 because GAP is also generated from fructose-1,6-bisphosphate via aldolase. Thus, germination may not be triggered merely by a decrease in GAP. An alternative possibility is that germination may be regulated by the balance between GAP and DHAP levels. In support of this hypothesis, we found that the inhibitory effect of GAP was alleviated by the addition of DHAP. Since mammalian/mechanistic TORC1 (mTORC1) is reported to be activated by DHAP in human cells (29), DHAP may be involved in the regulation of TORC1 in quiescent yeast cells. Overall, our results suggest that Tpi1 and its products are critical regulators for switching between positive and negative regulating pathways for germination. Exit from the stationary state may be similarly regulated. However, further analyses are required to clarify the regulations and roles of these molecules during exit from quiescent states.

We demonstrated that exit from the stationary state in C. glabrata is also manipulated by GAP and heat, suggesting that yeast cells share the same mechanism to maintain the quiescent state. Intriguingly, however, growth of C. glabrata stationary cells was induced when the cells were suspended in water but not in YPAD medium. Thus, for C. glabrata, exit from the quiescent state may be suppressed by another molecule included in YPAD media. In the present study, we assessed S. cerevisiae and C. glabrata cells, but it is tempting to hypothesize that the dormant state is generally suppressed via negative regulatory pathways, although the detailed mechanism may differ between species. In particular, a negative regulatory mechanism would be required for other ascospores and endospores to prevent premature germination. Indeed, in Bacillus anthracis spores, an alanine racemase present in the exosporium was reported to be required for preventing premature germination since the enzyme can convert germinant l-alanine to d-alanine (30). It is also notable that, in spores of some filamentous fungi or bacteria, germination is known to be activated by heat treatment (31–33). As with S. cerevisiae spores, this phenomenon could be interpreted by abrogation of the negative regulation process. Spores and stationary cells are resistant to stresses and antibiotics, which is problematic for the treatment of infectious diseases (34, 35) and food poisoning (36, 37). We demonstrated that dormancy in yeast cells can be manipulated by modulating the negative regulatory process. Thus, this regulatory process would be an attractive target to improve antibiotic treatments.

In the present study, we focused on the molecular basis of heat-induced germination, and thus, the physiological role of this phenomenon remains unknown. However, it is intriguing that germination is induced by incubation at 40°C for 1 h because yeast cells could experience such conditions in nature, for example, when spores are eaten by animals with high body temperature. Thus, this property may be beneficial for S. cerevisiae to survive in nature.

## MATERIALS AND METHODS

### Strains.

The genotypes and sources of yeast and E. coli strains used in this study are listed in Table S2 in the supplemental material. The S. cerevisiae strains used in this research were the rapidly sporulating SK-1 background strains AN120 (diploid) and AN117-16D (haploid) (38). To create the *CLB2-GFP*-expressing strain, pFA6a-GFP(S65T)-HIS3MX6 was amplified by PCR using primers clb*2*-F2 and clb*2*-R1 and then inserted at the 3′ end of the *CLB2* coding sequence locus of haploid segregants of AN120 (38) by homologous recombination. The haploid cells were mated to generate diploid cells. The C. glabrata strain used in this study was KUE100 (39). E. coli BL21(DE3) (Invitrogen) was used to produce recombinant Tpi1. To create the *tpi1Δ* strain, CRISPR/Cas9-mediated genome engineering procedure was used. The 1,000-bp fragments of *TPI1* upstream and downstream sequences were amplified by using primers tpi1-up-F/R and tpi1-down-F/R. The genomic DNA of AN120 was used as the template. These DNA fragments were linked by overlapping PCR and used as the DNA donor. The DNA donor was transformed into AN120 together with p414-TEF1p-Cas9-CYC1t and p426-SNR52p-gRNA-TPI1. *tpi1Δ* cells were selected by colony PCR using the primers VF and VR. The mutant cells were cultured on synthetic dextrose (SD) (3% ethanol and 0.1% glucose) plates supplemented with 1 mg/mL 5-fluoroanthranilic acid or 1 mg/mL 5-fluoroorotic acid to remove p414-TEF1p-Cas9-CYC1t and p426-SNR52p-gRNA-TPI1.

### Plasmids.

All the oligonucleotides and plasmids used in this study are listed in Tables S3 and S4, respectively. The S. cerevisiae *TPI1* gene was amplified by PCR using AN120 genomic DNA as a template. P. woesei *TPIA* (GenPept accession no. CAA70690) optimized for expression in yeast was synthesized by Talen-Bio Scientific. These genes were cloned into pRS316-TPI1pr-FLAG for expression in S. cerevisiae cells. To construct pRS316-TPI1pr-FLAG, the *TPI1* promoter was first amplified by PCR using R5 and R6 as primers and AN120 genomic DNA as the template and then ligated into SacI/SpeI sites of pRS426TEF2-FLAG (40). From the resulting plasmid, a gene fragment containing the *TPI1* promoter, three tandem FLAG repeats, and the *ADH1* terminator was digested out by SacI and KpnI. The DNA fragment was cloned into pRS316 (41). pRS316-TPI1pr-FLAG was used as a control (empty) vector. To produce recombinant proteins in E. coli, pET28a (Novagen) was used as the parental plasmid. Mutations were introduced with PCR-based site-directed mutagenesis. Plasmids p414-TEF1p-Cas9-CYC1t and p426-SNR52p-gRNA were purchased from Addgene for CRISPR/Cas9-mediated gene disruption. The 20-bp protospacer sequence (GAATTGAGAATCTTATACGG) of guide RNA (gRNA), which can recognize the *TPI1* gene used in this study, was predicted by the website http://www.e-crisp.org/E-CRISP/index.html. p426-SNR52p-gRNA-TPI1 was used to express the gRNA targeting *TPI1*. This plasmid was generated by PCR in that p426-SNR52p-gRNA was amplified with primers gRNA-F and gRNA-R. The sequences of the oligonucleotides used in this study are listed in Table S3.

### Yeast culture and sporulation.

Unless otherwise noted, yeast cells were cultured in YPAD liquid medium (1% yeast extract, 2% peptone, 2% dextrose, and 0.003% adenine). The *tpi1Δ* cells were cultured in YPAD medium supplemented with 3% ethanol and 0.1% glucose. Sporulation was performed as described previously (42). Briefly, yeast cells derived from a single colony were grown overnight in 5 mL of YPAD liquid medium, and then 5 mL of the culture was added to 200 mL of YPAce medium (1% yeast extract, 2% peptone, 2% potassium acetate, and 0.003% adenine) and grown for 24 h. The cells were harvested by centrifugation, washed with sterilized water, resuspended in 100 mL of 2% potassium acetate medium, and cultured for 24 h.

### Preparation of spores.

To release spores from asci, the asci were suspended in 5 mL of 1.2 M sorbitol solution, and 20 μL of lyticase (Sigma-Aldrich) stock solution (10,000 U/mL, dissolved in 500 μL of 50% glycerol) was added. After 3 h of incubation at 37°C, the spores were washed six times with 0.5% Tween 20 and 2 times with water.

### Preparation of ascal and cell lysates.

Yeast spores cultured for 24 h in potassium acetate medium were used to prepare ascal lysate. Vegetative yeast cells cultured overnight in YPAD were used to prepare vegetative cell lysates. Two grams (wet weight) of asci or vegetative cells were suspended in 10 mL of sterilized water and sonicated on ice with a probe-type sonicator (Sonics). The sonication conditions were as follows: power, 40%, and duration, 40 min, with cycles of 5 s on and 2 s off. Spores or debris were removed by centrifugation (15,000 × *g*, 30 min), and the supernatant was used for experiments.

### Germination assay with CFW staining.

To treat ascal lysate with heat, 1 mL of the lysate was incubated in a metal bath (Allsheng) at 30 to 95°C for 10 min. To prepare proteinase-treated ascal lysate, 2 μL of 20 mg/mL proteinase (Sigma-Aldrich) solution was added to 1 mL of the lysate, and the lysate was incubated at 37°C for 1 h. To prepare chloroform-treated ascal lysate, 1 mL of the lysate was mixed with 1 mL of chloroform and then centrifuged to collect supernatant. To treat the ascal lysate with anion or cation exchange resins, AG 50W-X8 or AG 1-X2 resins (Bio-Rad) were preincubated with 1 M NaCl and then washed with water. One milliliter of ascal lysate was incubated with 50 mg of resin at 4°C for 2 h with gentle shaking and then centrifuged to collect the supernatant. For the germination assay using ascal lysate, 20 mg (wet weight) of spores was suspended in 100 μL of ascal lysate and incubated at 30°C for 6 h. To treat asci or spores with heat, 20 μL of asci or spore suspensions (10^7^ cells/mL in water) was incubated in a metal bath at 30 to 95°C for 15 s to 1 h. The cell suspensions were incubated at 30°C for 6 to 36 h.

To quantify germination efficiency, the cell suspensions were centrifuged to remove the supernatant, washed twice with sterilized water, and then suspended in 100 μL of distilled water. Then, 10 μL of CFW (Sigma-Aldrich) solution (1 mg/mL) was added. The mixture was incubated at 30°C for 30 min, washed twice with sterilized water, and resuspended in 50 μL of distilled water. The cells were observed under a fluorescence microscope, and those stained with CFW were counted.

### Western blot analysis.

To extract the proteins from the cells, the cells (20 mg) were suspended in 200 μL of 8 M urea sonicated with a probe-type sonicator on ice. The sonication conditions were as follows: power, 45%, and duration, 60 min, with cycles of 1 min on and 1 min off. The protein concentration was measured with a NanoDrop spectrophotometer (Thermo Scientific), and 100 μg of protein was subjected to 10% SDS-PAGE. The proteins were transferred to polyvinylidene difluoride (PVDF) membranes (Bio-Rad, Shanghai). Mouse anti-FLAG (Transgen), mouse anti-GFP (Transgen), or anti-actin antibody (Novus) was used as the primary antibody at a 1:5,000 dilution. Goat anti-mouse IgG-horseradish peroxidase (HRP; Transgen) was used as a secondary antibody at a 1:5,000 dilution. Clarity Western ECL substrate (Bio-Rad) was used to display the bands, and Image Quant LAS 4000 (GE Healthcare) was used to acquire the images.

### Production and purification of recombinant Tpi1 in E. coli.

The plasmids pET28a-ScTPI1 and pET28a-ScTPI1^E165A^ were transformed into E. coli BL21(DE3) cells, and the transformants were cultured overnight in 5 mL of LB medium supplemented with 50 μg/mL kanamycin. Then, 1 mL of the overnight culture was transferred to 100 mL of LB medium supplemented with 50 μg/mL kanamycin and incubated at 37°C with shaking. When the optical density at 600 nm (OD_600_) of the cell culture was 0.8 to 1.0, isopropyl β-d-1-thiogalactopyranoside (IPTG) was added at a final concentration of 0.1 mM to induce Tpi1 expression, and the cell culture was incubated at 16°C for 20 h. Next, the cells were collected by centrifugation at 9,000 × *g* for 3 min, and the pellets were suspended in 10 mL of lysis buffer (25 mM Tris-HCl [pH 8.0] and 150 mM NaCl). The cells were lysed by sonication with a probe-type sonicator on ice. The sonication conditions were as follows: power, 45%, and duration, 40 min, with cycles of 5 s on and 2 s off. The supernatant was acquired by centrifugation at 9,000 × *g* for 30 min and loaded onto Ni-nitrilotriacetic acid (NTA) agarose (Cube Biotech). The Ni-NTA agarose was washed with lysis buffer, and then, His-tagged Tpi1 was eluted with elution buffer (25 mM Tris-HCl [pH 8.0], 150 mM NaCl, 250 mM imidazole, and 5% glycerol). To concentrate and desalt the purified Tpi1 protein, an Amicon Ultra filter (10 kDa) was used. The amount of protein was measured using a NanoDrop spectrophotometer (Thermo Scientific).

### Purification of the inhibitor.

AG 50W-X8 or AG 1-X2 resins were preincubated with 1 M NaCl and then washed with water. To purify the inhibitor, 5 mL ascal lysate was first incubated with 250 mg of cation exchange resin at 4°C for 2 h with gentle shaking. After the cation exchange resin was removed by centrifugation, the lysate was incubated with 250 mg anion exchange resin at 4°C for 2 h with gentle shaking. The anion exchange resin was packed in a 0.5-cm inner diameter affinity chromatography column (Beyotime) and washed with 20 mM Tris-HCl (pH 8.0). Proteins were eluted with 20 mM Tris-HCl (pH 8.0) supplemented with 10 to 300 mM NaCl. The eluted fractions were dialyzed against water three times for 48 h, followed by lyophilization. The lyophilized fractions were suspended in 100 μL of water, and 10 μL of each suspension was subjected to 10% SDS-PAGE. The gel was stained with Protein Fast silver stain kit (Beyotime) according to the manufacturer’s instructions. To analyze inhibitory activity, 50 μL of the suspension was mixed with 50 μL of 0.4% glucose solution, and 20 mg of spores was added. After incubation at 30°C for 6 h, germination efficiency was determined by CFW staining.

### In-gel digestion and liquid chromatography-mass spectrometry analysis.

The protein band was cut into small pieces, placed in the tube (mass spectroscopy grade; Axygen), and washed with water 5 times. For destaining, 200 μL of 100 mM ammonium bicarbonate/acetonitrile (1:1 [vol/vol]) was added, and the mixture was incubated for 30 min with occasional vortexing. Then, 500 μL of pure acetonitrile was added until the gel pieces became white and shrank. After removal of acetonitrile, 200 μL of 100 mM ammonium bicarbonate with 10 mM dithiothreitol was added, the mixture was incubated at 37°C for 1 h 20 min, and the gel was shrunk with acetonitrile. Then, 200 μL of 100 mM ammonium bicarbonate with 10 mM iodoacetamide was added, the suspension was incubated in the dark for 50 min, and 200 μL acetonitrile was added to shrink the gel. After the gel had completely shrunk, 2 μL of trypsin solution (mass spectroscopy grade; Shuangmo) was added, and the mixture was incubated at 37°C overnight with gentle shaking. To extract peptides, 100 μL of extraction buffer (1:2 [vol/vol] 5% formic acid/acetonitrile) was added, and the suspension was incubated at 37°C for 15 min in a shaker. Then, the sample was cleaned up using a C_18_ stage tip (3M) and lyophilized.

The lyophilized peptides were resuspended in 10 μL 2% acetonitrile and 0.1% formic acid solution and then analyzed using an EASY-nLC 1200 system (Thermo Scientific) coupled with a high-resolution Orbitrap Fusion Lumos spectrometer (Thermo Scientific). The injection volume for each sample was 3 μL. The samples were first separated on an EASY-nLC 1200 system in a rapid-separation liquid chromatography (RSLC) C_18_ column (1.9 μm by 100 μm by 20 cm) packed in-house. All samples were subjected to two LC-MS/MS runs on an Orbitrap Fusion Lumos mass spectrometer. Data-dependent high-energy collisional dissociation (HCD) fragmentation was performed on the top 20 most abundant ions. For global peptide analysis, a mobile phase consisting of water (A) and 0.1% formic acid and 90% acetonitrile (B) was used with the following elution gradient: 3 to 8% B, 8 min; 8 to 28% B, 80 min; 28 to 32% B, 22 min; 32 to 80% B, 5 min; and 80% B, 5 min. The flow rate was maintained at 450 nL/min. The spray voltage was in static mode. Spectra (automatic gain control [AGC] target of 4 × 10^5^ and maximum injection time of 50 ms) were collected from 350 to 2,000 *m/z* at a resolution of 60,000, followed by data-dependent HCD MS/MS (at a resolution of 30,000; HCD collision energy, 34%; stepped collision energy, 5%; AGC target, 5 × 10^4^; maximum injection time, 35 ms; and microscans, 1). Charge state screening was enabled to reject singly charged ions and ions with more than eight charges. A dynamic exclusion time of 45 s was used to discriminate newly selected from previously selected ions.

### Identification of inhibitors.

The acquired MS/MS spectra were analyzed with MaxQuant software against the protein database from S. cerevisiae strain S288c (ATCC 204508) downloaded from UniProt website (http://www.uniprot.org/) updated in September 2020. The parameters were set as default if not otherwise stated. The enzyme specificity was set to trypsin/P (the C terminus of Arg or Lys with cleavage at the proline bond allowed), and the maximum number of missed cleavage sites was set as two. Carbamidomethyl (C, 43.028) was set as the fixed modification. Deamidation (N, 29.018) was set as the variable modifications. Proteins and peptides were identified using a target-decoy approach in revert mode and quantified using intensity data (peak area from extracted ion chromatography) using the Andromeda search engine integrated into the MaxQuant environment. MaxQuant search was performed with a fragment ion mass tolerance of 0.02 Da and a parent ion tolerance of 10.0 ppm. The false-discovery rates (FDRs) of protein groups and peptides were less than 0.01.

### Detection of glucose in ascal lysate.

To detect glucose by HPLC, the ascal lysate was filtered by a 0.22-μm filtration membrane (Millipore) and analyzed by HPLC (Waters) equipped with Waters Sugar-Pak column (300 mm by 6.5 mm). EDTA calcium salt (500 mg/L) was used as mobile phase at a flow rate of 0.5 mL/min. The column temperature was 80°C, and a refractive index detector was used. To quantify glucose in ascal lysates, glucose standard solutions (0.5, 1, 2, 5, 10, and 20 mg/mL) were prepared. These standards were subjected to HPLC, and a calibration curve was generated by plotting the peak area (Fig. S11a). The concentration of glucose in the samples was determined based on this calibration curve.

To detect glucose by a chemical method, a glucose test kit (Rongsheng) was used according to the manufacturer’s instructions.

### Detection and quantification of DHAP and GAP.

Samples were run in Sciex LC with Acquity ultraperformance liquid chromatograph (UPLC) BEH Amide 1.7-μm (2.1 by 100 mm) column (Waters) coupled to an AB Sciex 5500 triple-quadrupole trap mass spectrometer (Q Trap MS) with electrospray ionization (ESI) in negative mode (Waters). Instrument control and data integration were performed using Analyst software version 1.6.2 (AB Sciex). The mobile phase was a mixture of two solvents, A (20 mM ammonium formate, 90% acetonitrile, and 0.05% formic acid) and B (20 mM ammonium formate, 60% acetonitrile, and 0.05% formic acid). The optimized linear gradient system was as follows: 0 min, 15% A; 0 to 0.5 min, to 15% A; 0.5 to 5 min, to 95% A; 5 to 7 min, to 95% A; and 7 to 10 min, 15% A. The autosampler was set to 4°C. The injection volume was 2 μL, and the flow rate was 300 μL/min. The injection needle was washed with 50% methanol after each injection. The column oven was 45°C. The instrument parameters were set as follows: ion spray voltage (IS), −4,500 V; temperature, 550°C; ion source gas (GS1), 60 lb/in^2^; ion source gas (GS2), 60 lb/in^2^; curtain gas (CUR), 35 lb/in^2^; and collision gas (CAD), medium.

DHAP and GAP have the same precursor and product ions. The major MS/MS fragment patterns of DHAP and GAP were determined by multiple-reaction monitoring (MRM). After ionization, each precursor ion was isolated in the first quadrupole (Q1) and collided with gas in Q2; then, the fragment ions were detected in Q3 (MS/MS experiments). The MRM transition (Q1/Q3) settings for DHAP and GAP are shown in Fig. S6.

To quantify DHAP and GAP, DHAP and GAP standard solutions (Sigma-Aldrich) with different concentrations (0.1, 0.5, 5, 20, and 50 μg/mL) were prepared with 20% methanol. Standard curves were generated based on linear regression of the analyte response (*y*) versus analyte concentration (*x*) using *m/z* 97 as quantifying ion (Fig. S11b). To analyze DHAP and GAP levels secreted from spores, 20 mg (wet weight) of spores was incubated in 100 μL of 0.2% glucose solution or water (control) for 1 h at 30°C. The supernatant of the spore suspension was collected by centrifugation. After the addition of 400 μL of cold methanol solution, the sample was mixed with vortex for 1 min and sonicated with an ultrasonic cleaner (Sonics, Kunshan). The sonication conditions were as follows: power, 60%, and duration, 15 min. Then, the sample was centrifuged at 12,000 × *g* at 4°C for 15 min. An aliquot of the supernatant was divided into two new tubes and lyophilized. The lyophilized samples were suspended in 100 μL of 20% methanol. The concentrations of DHAP and GAP were determined based on this calibration curve (Fig. S11c).

### Preparation of cells in the stationary- and logarithmic growth phases.

Stationary S. cerevisiae cells were prepared as described previously (43). Cells from single colonies were cultured in 5 mL YPAD at 30°C for 12 h. Then, the culture was added to 50 mL YPAD to obtain an initial OD_660_ of 0.2. The flask was then incubated at 30°C with shaking. The OD_660_ was measured every 24 h, and cells were cultured until growth stopped completely. Stationary C. glabrata cells were similarly prepared. Stationary *tpi1Δ* cells were prepared in YPAD medium supplemented with 3% ethanol and 0.1% glucose. The incubation times for S. cerevisiae and C. glabrata were 13 to 14 days and 5 to 8 days, respectively. To prepare S. cerevisiae cells in logarithmic growth phase, the cells were grown in 5 mL YPAD at 30°C overnight, and then, 0.1 mL was inoculated into 5 mL of fresh YPAD. The cells were cultured at 30°C with shaking for 4 to 6 h to reach an OD_660_ of 1.0.

### Growth assays of stationary and logarithmic cells.

Stationary S. cerevisiae cells were treated with heat at 40°C for 1 h, and C. glabrata stationary cells were treated with heat at 40°C for 1 h or 95°C for 15 s. The heated cells were transferred to 10 mL of fresh liquid YPA (glucose free), YPAD (0.02% glucose), YPAD (0.03% ethanol and 0.02% glucose), or water supplemented with 0.02% glucose to obtain an initial OD_660_ of 0.1 to 0.2. The cells were cultured at 30°C, and the OD_660_ was measured every 20 min or 2 h. To assess the growth rates of stationary-phase cells in the presence of GAP, the cells were transferred to 10 mL of fresh liquid YPAD medium supplemented with 1% glucose to obtain an initial OD_660_ of 0.1 to 0.2. The cells were cultured at 30°C. GAP (0.1 μmol) was added to the culture every 20 min, and the OD_660_ was measured. Cell number was quantified with either turbidity (OD_660_) or a platelet counter. To draw growth curves of *tpi1Δ* cells, the mutant cells were precultured in YPAD (3% ethanol and 0.1% glucose) media to an OD_660_ of 1.0 and then transferred to 10 mL of fresh liquid YPAD (3% ethanol and 0.1% glucose) to obtain an initial OD_660_ of approximately 0.2, and the OD_660_ was measured every 2 h.

### Microscopy.

Microscopy images were obtained using a Nikon C2 Eclipse Ti-E inverted microscope with a DS-Ri camera equipped with NIS-Elements AR software.

### Statistical analysis.

All experiments were performed with at least three independent samples. All statistical tests were performed with GraphPad Prism 8 (GraphPad Software). Statistical significance was determined by two-tailed unpaired Student’s *t* tests, and *P* values of <0.05 were considered to be statistically significant.

### Data availability.

The data supporting the findings of this study are available within the article and its supplemental material. Further relevant data are available from corresponding authors upon reasonable request.
