# Supplementary material for: Triosephosphate Isomerase and Its Product Glyceraldehyde-3-Phosphate Are Involved in the Regulatory Mechanism That Suppresses Exit from the Quiescent State in Yeast Cells
Source: Microbiol Spectr. 2022 Aug 4;10(4):e00897-22. doi: 10.1128/spectrum.00897-22 (PMC9430402; doi:10.1128/spectrum.00897-22)
Supplement: Supplemental file 1 — Supplemental material. Download spectrum.00897-22-s0001.pdf, PDF file, 2.6 MB [file spectrum.00897-22-s0001.pdf]

Fig. S1

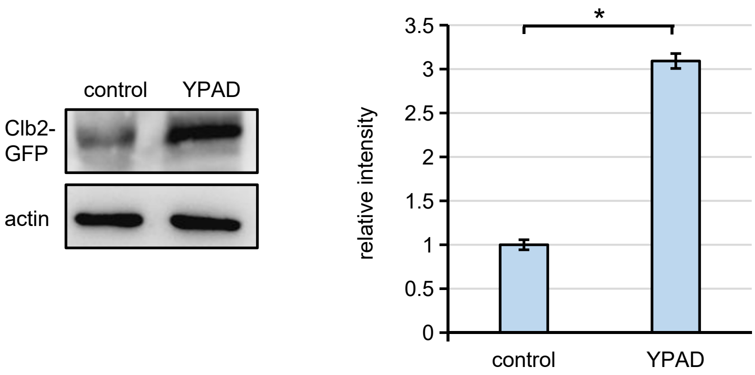

FIG S1 The expression levels of Clb2 increase in germinating cells. Left panel: Cells expressing Clb2-GFP from the *CLB2* promoter were allowed to sporulate, and the spores released form asci were incubated in YPAD at 30°C for 6 h. Clb2-GFP was detected in their lysate by western blotting analysis using an anti-GFP antibody. As a control, Clb2-GFP was detected in spores before incubation in YPAD. Actin was detected as loading control. Right panel: Relative intensities of Clb2-GFP signals detected in the western blotting analysis. Clb2-GFP detected in spores before incubation in YPAD (control) was taken as 1. The data are presented as the mean ± SE, n=3. Statistical analysis was performed by two-tailed unpaired Student's *t* tests, *, $P < 0.05$.

Fig. S2

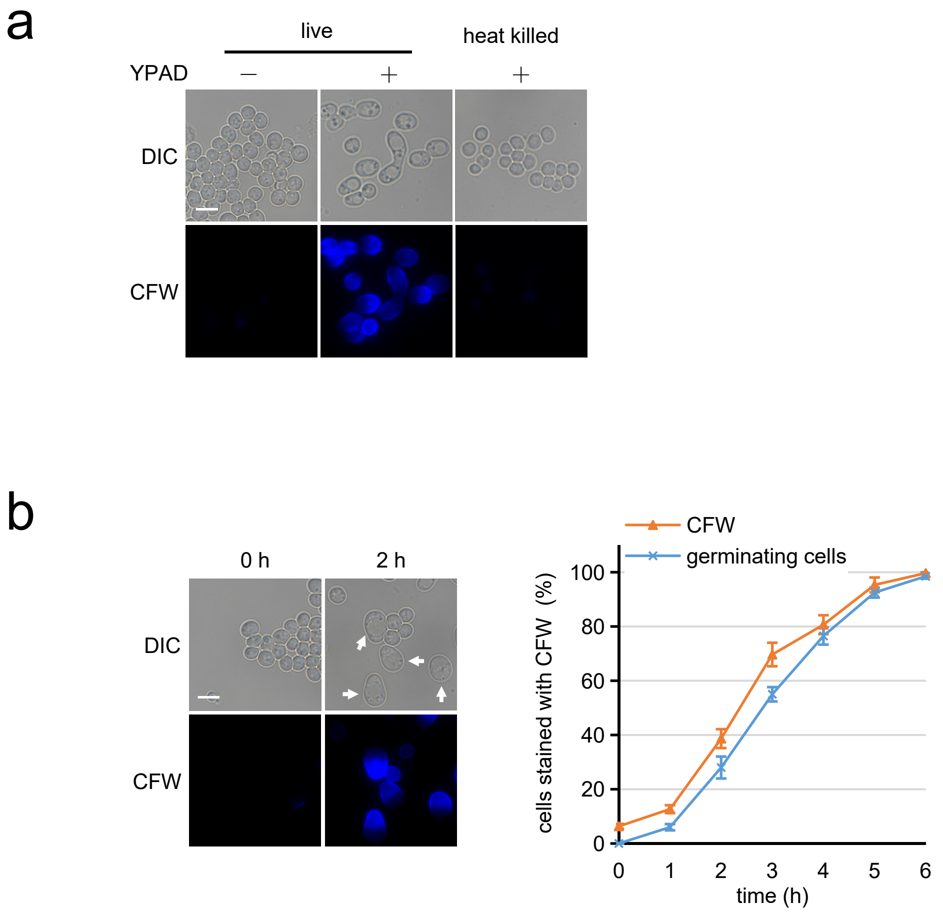

FIG S2 Germination levels can be assayed with CFW staining. (a), Live or heat killed spores were incubated with (+) or without (-) YPAD media for 6 h at 30°C, and they were stained with CFW. Representative images obtained with differential interference contrast (DIC) microscopy and fluorescence microscopy (CFW) images are shown. Scale bar, 5 µm. (b), Left panels: Spores were incubated YPAD media for 2 h at 30°C, and stained with CFW. Representative images obtained with differential interference contrast (DIC) microscopy and fluorescence microscopy (CFW) images are shown. Scale bar, 5 µm. Arrows indicate germinating spores. Right panel: Spores were incubated with YPAD medium at 30°C for indicated times and stained with CFW. The percentage of CFW stained spores (CFW) and germinating cells were measured over time. Swollen and deformed spores as indicated arrows in left panels are defined as germinating spores. 300 spores were analyzed for each assay. The data are presented as the mean ± SE, n=3 (b).

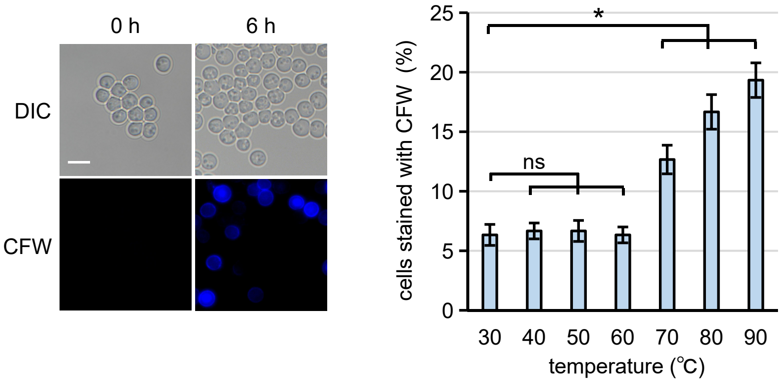

FIG S3 Spore germination is induced by heat treatment. Left panels: Spores released from asci were suspended in water. The spore suspension was treated with heat at 30°C or 90°C for 15 sec. After incubation at 30°C for 6 h, spores were stained with CFW. Representative images obtained with differential interference contrast microscopy (DIC) and fluorescence microscopy (CFW) are shown. Scale bar, 5 µm. Right panels: Spores suspended in water were treated with heat at indicated temperatures for 15 sec. The suspension was subsequently incubated at 30°C for 6 h. Spores were stained with CFW to assay germination efficiency. 300 spores were analyzed for each assay.The data are presented as the mean ± SE, n=3. Statistical analysis was performed by two-tailed unpaired Student's *t* tests, *, $P < 0.05$; ns, not significant.

Fig. S4

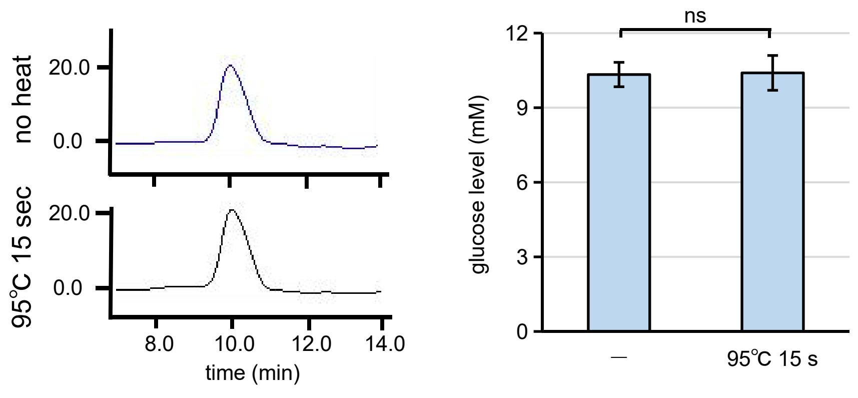

FIG S4 Detection of glucose in ascal lysate. Left panels: Glucose in ascal lysate treated with or without heat (95°C, 15 sec) was detected by HPLC. Right panel: The glucose concentration in ascal lysate treated with or without heat (95°C, 15 sec) was measured by HPLC. The data are presented as the mean ± SE, n=3. Statistical analysis was performed by two-tailed unpaired Student's *t* tests, ns, not significant.

Fig. S5

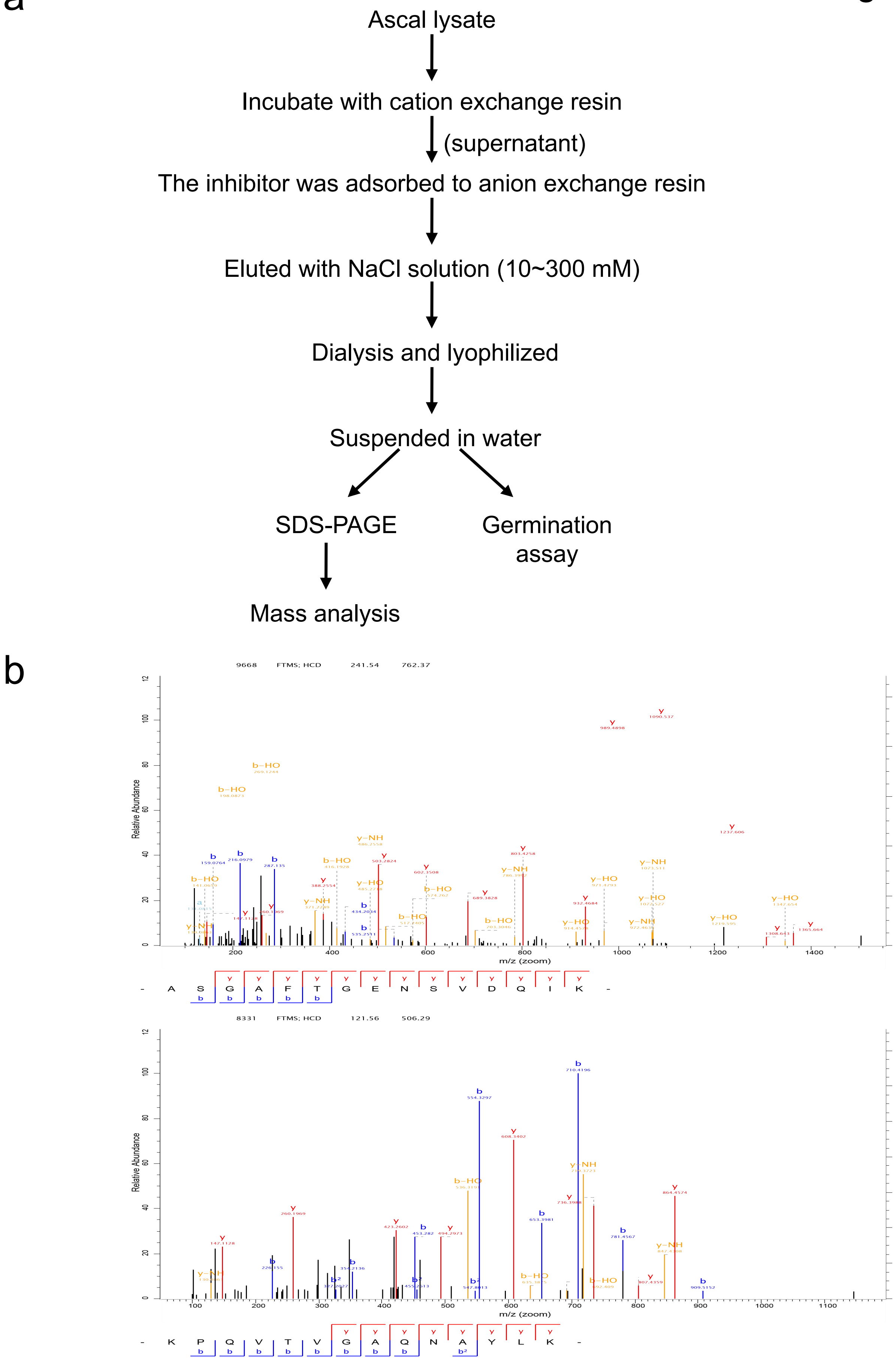

FIG S5 Identification of the inhibitor present in the cytosolic ascal. (a), The procedure to purify the inhibitor present in the ascal cytosol. (b), MS/MS fragments of peptides with m/z 506.29 and 762.37 from the purified inhibitor are shown.

Fig. S6

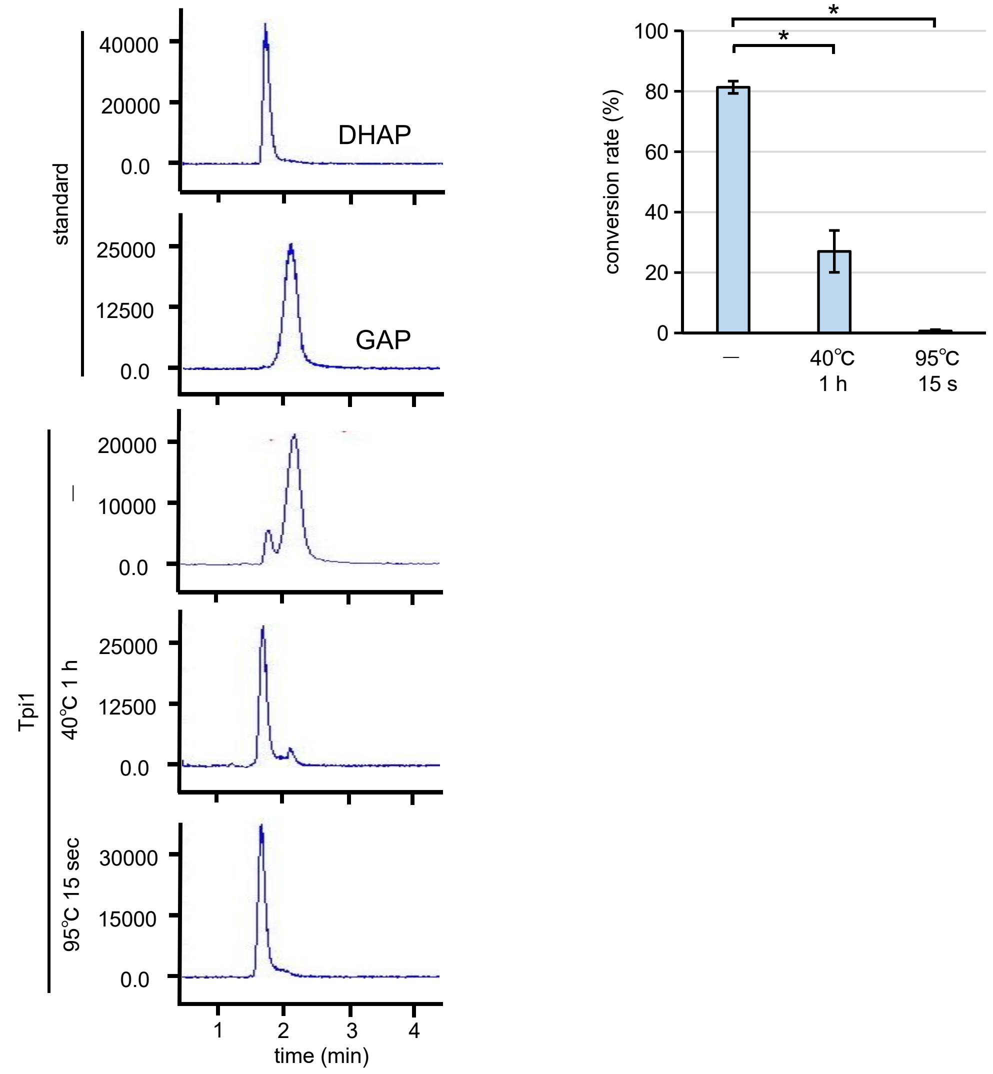

FIG S6 In vitro activity assay of recombinant Tpi1. Left panels: Recombinant Tpi1 treated with or without (-) heat (40°C, 1 h or 95°C, 15 sec) were incubated with DHAP at 30°C for 2 h. The reaction mixtures were subjected to HPLC to detect DHAP and GAP. DHAP and GAP were subjected to HPLC as standard samples. Right panel: Conversion efficiency from DHAP to GAP mediated by Tpi1 (-) or heat inactivated (40°C, 1 h or 95°C, 15 sec) Tpi1. The data are presented as the mean ± SE, n=3. Statistical analysis was performed by two-tailed unpaired Student's *t* tests, *, $P < 0.05$.

Fig. S7

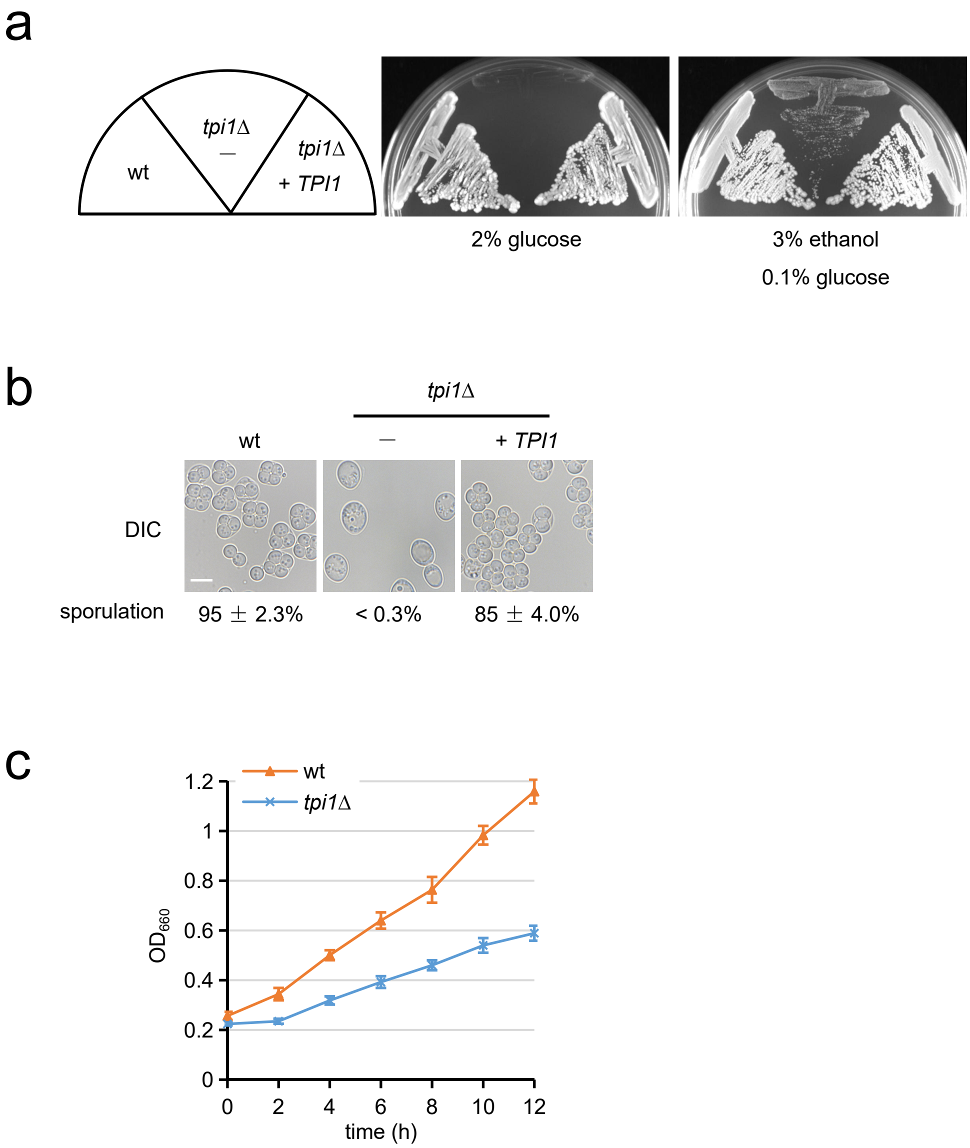

FIG S7 Characterization of *tpi1*Δ cells. (a), Wild-type (wt) or *tpi1*Δ cells harboring the *TPI1* expression plasmid (+ *TPI1*) or empty plasmid (-) were streaked on YPAD *pl*ate supplemented with 2% glucose or 3% ethanol and 0.1% glucose. (b), Wild-type (wt) or *tpi1*Δ cells harboring the *TPI1* expression plasmid (+ *TPI1*) or empty plasmid (-) were sporulated and observed by bright-field microscopy. Bar, 5 µm. Their sporulation efficiencies are shown under the images. Data presented are the mean ± SE of three independent samples (more than 300 cells were analyzed for each assay). (c), Wild-type (wt) or *tpi1*Δ cells were cultured in YPAD supplemented with 3% ethanol and 0.1% glucose at 30°C. Cell growth was assayed by measuring turbidity. The data are presented as the mean ± SE, n=3.

Fig. S8

a

DHAP/GAP specific parameters

Q1	Q3	DP (V)	CE (eV)
169	97	-35	-12
169	79	-35	-32

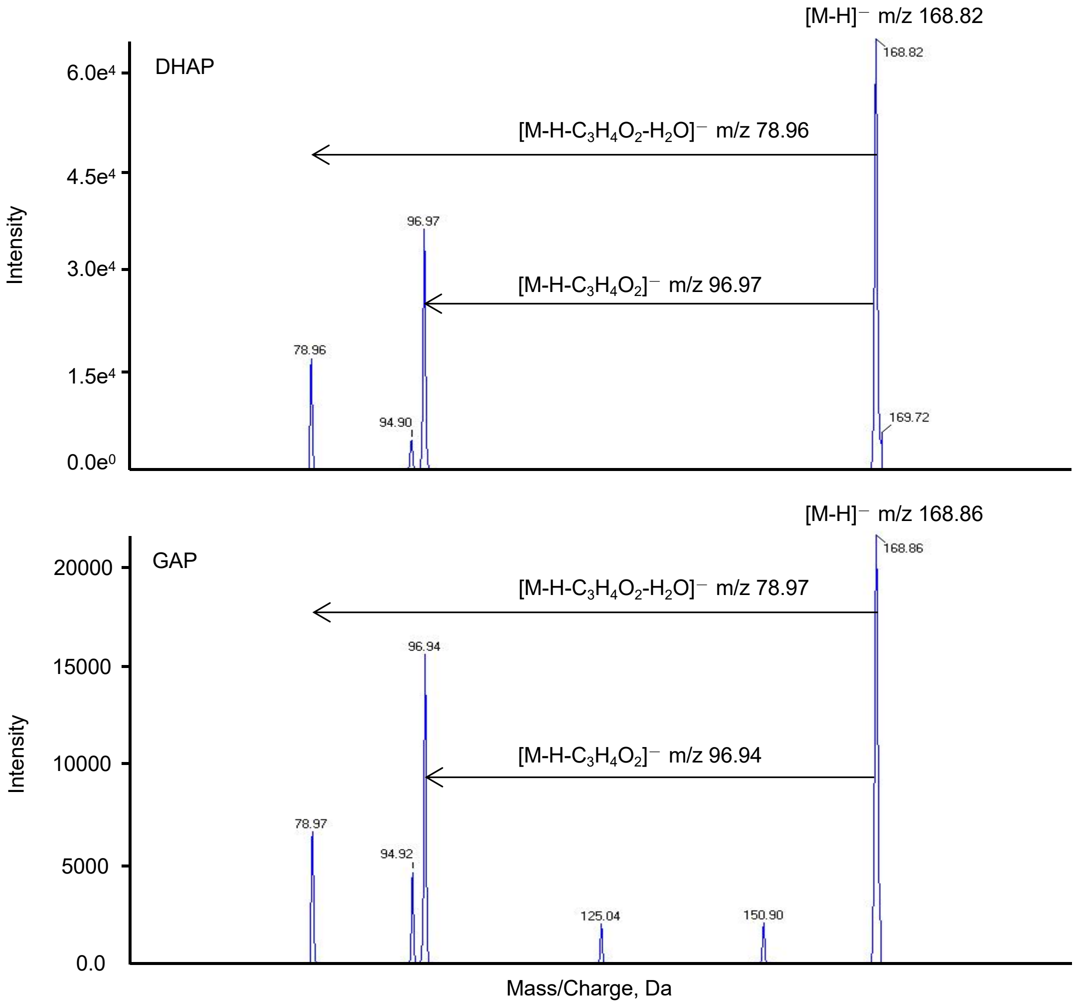

b

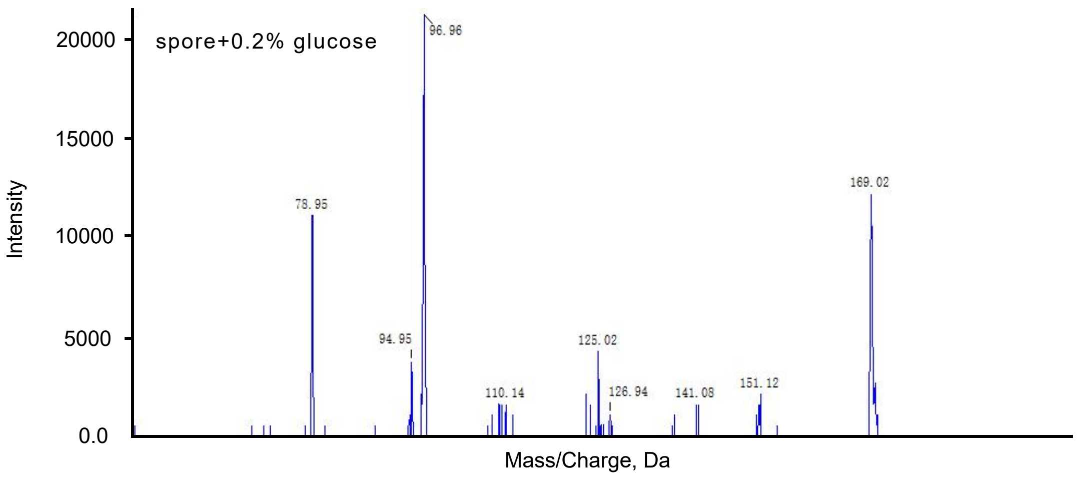

FIG S8 DHAP is secreted from spores incubated in glucose. (a), Calibration solutions containing 1 μg/ml DHAP and GAP were used to determine LC-MS/MS fragmentation patterns. DHAP generated a m/z 97 fragment in the collision quadrupole (Q2), which corresponded to the loss of the carbohydrate moiety in the collision cell. (b), Spores were incubated with 0.2% glucose solution or water (control) at 30°C for 1 h. Supernatants of the spore suspensions were subjected to LC-MS. MS analysis was performed for DHAP peak detected by liquid chromatography shown in Fig. 4c.

Fig. S9

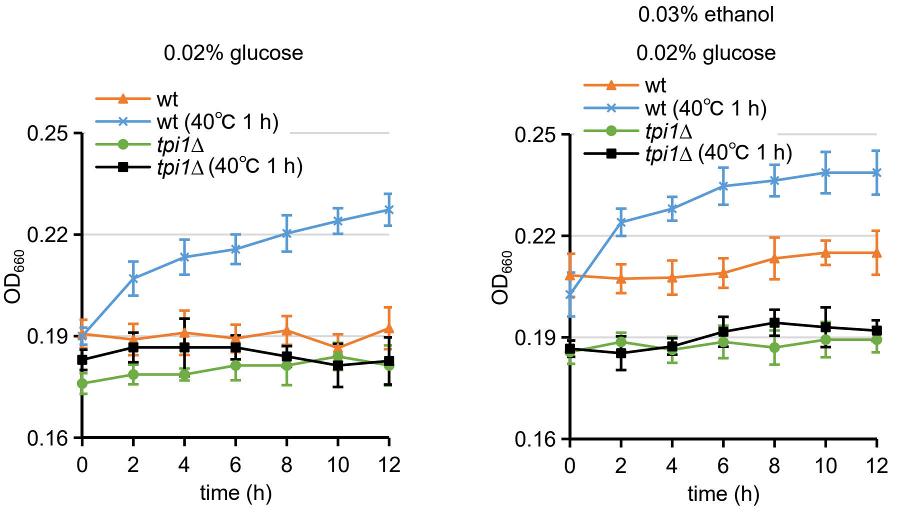

FIG S9 Stationary *tpi1*Δ cells do not grow in the low carbon source medium. Wild-type (wt) or *tpi1*Δ cells treated with or without heat (40°C, 1 h) were cultured in YPAD supplemented with 0.02% glucose (left panel) or 0.03% ethanol and 0.02% glucose (right panel) at 30°C for 12 h. Cell growth was assayed by measuring turbidity. The data are presented as the mean ± SE, n=3.

Fig. S10

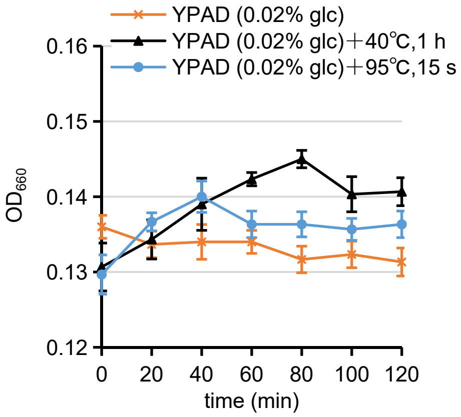

FIG S10 Stationary state of *C. glabrata* cells is not released by heat in YPAD medium. Stationary *C. glabrata* cells treated with or without heat (40°C, 1 h or 95°C, 15 sec) were incubated in YPAD (0.02% glucose) and cells were incubated at 30°C for 2 h. Cell growth was assayed by measuring turbidity. The data are presented as the mean ± SE, n=3.

Fig. S11

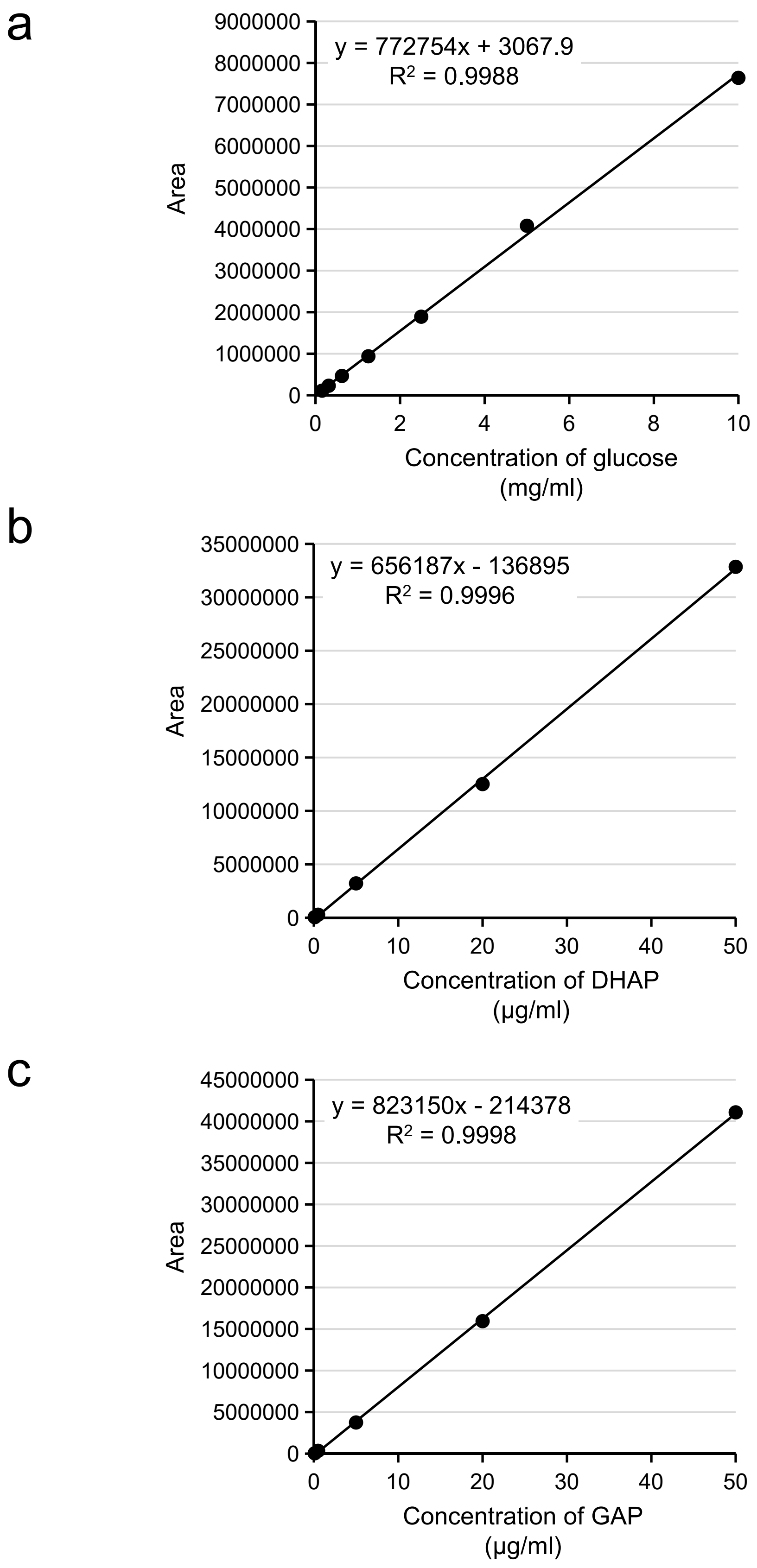

FIG S11 Calibration curves of glucose, DHAP and GAP. Glucose, DHAP and GAP standard solutions with different gradients were prepared. Standard curves are generated based on linear regression of the analyte response (y) versus analyte concentration (x).

TABLE S1 Analysis of Mass Spectrometry Data.

Protein IDs	Peptide counts	Fasta headers	Sequence coverage [%]	Mol. weight [kDa]	Score	Intensity	MS/MS count
sp|P00942|TPIS_YEAST	10	sp|P00942|TPIS_YEAST Triosephosphate isomerase OS=Saccharomyces cerevisiae (strain ATCC 204508 / S288c) OX=559292 GN=TPI1 PE=1 SV=2	46	26.795	323.31	2212100000	25
sp|P47089|DENR_YEAST	1	sp|P47089|DENR_YEAST Translation machinery-associated protein 22 OS=Saccharomyces cerevisiae (strain ATCC 204508 / S288c) OX=559292 GN=TMA22 PE=1 SV=1	3.5	22.495	74.737	72392000	1
sp|Q06132|SGD1_YEAST	1	sp|Q06132|SGD1_YEAST Suppressor of glycerol defect protein 1 OS=Saccharomyces cerevisiae (strain ATCC 204508 / S288c) OX=559292 GN=SGD1 PE=1 SV=1	0.7	102.85	12.685	0	2
sp|Q03124|RSC9_YEAST	2	sp|Q03124|RSC9_YEAST Chromatin structure-remodeling complex subunit RSC9 OS=Saccharomyces cerevisiae (strain ATCC 204508 / S288c) OX=559292 GN=RSC9 PE=1 SV=1	4.6	65.217	12.432	47969000	2
sp|P49167|RL38_YEAST	1	sp|P49167|RL38_YEAST 60S ribosomal protein L38 OS=Saccharomyces cerevisiae (strain ATCC 204508 / S288c) OX=559292 GN=RPL38 PE=1 SV=1	14.1	8.8264	9.7732	0	2
sp|P49687|NU145_YEAST	1	sp|P49687|NU145_YEAST Nucleoporin NUP145 OS=Saccharomyces cerevisiae (strain ATCC 204508 / S288c) OX=559292 GN=NUP145 PE=1 SV=1	0.8	145.66	9.7732	0	1
sp|P38877|CTF8_YEAST	1	sp|P38877|CTF8_YEAST Chromosome transmission fidelity protein 8 OS=Saccharomyces cerevisiae (strain ATCC 204508 / S288c) OX=559292 GN=CTF8 PE=1 SV=1	4.5	15.169	9.3739	0	2
sp|P40469|MET18_YEAST	1	sp|P40469|MET18_YEAST DNA repair/transcription protein MET18/MMS19 OS=Saccharomyces cerevisiae (strain ATCC 204508 / S288c) OX=559292 GN=MET18 PE=1 SV=1	0.6	117.88	9.3739	0	1
sp|P33299|PRS7_YEAST	1	sp|P33299|PRS7_YEAST 26S proteasome regulatory subunit 7 homolog OS=Saccharomyces cerevisiae (strain ATCC 204508 / S288c) OX=559292 GN=RPT1 PE=1 SV=1	1.9	51.982	9.2197	18416000	1
sp|P36004|KKQ8_YEAST	1	sp|P36004|KKQ8_YEAST Probable serine/threonine-protein kinase KKQ8 OS=Saccharomyces cerevisiae (strain ATCC 204508 / S288c) OX=559292 GN=KKQ8 PE=1 SV=2	1	82.589	9.0412	0	2
sp|P34247|UTP11_YEAST	1	sp|P34247|UTP11_YEAST U3 small nucleolar RNA-associated protein 11 OS=Saccharomyces cerevisiae (strain ATCC 204508 / S288c) OX=559292 GN=UTP11 PE=1 SV=2	3.6	29.735	8.8982	0	1
sp|P39980|SIT1_YEAST	1	sp|P39980|SIT1_YEAST Siderophore iron transporter 1 OS=Saccharomyces cerevisiae (strain ATCC 204508 / S288c) OX=559292 GN=SIT1 PE=3 SV=1	3.8	70.561	7.4065	312100	0
sp|P47164|MET7_YEAST	1	sp|P47164|MET7_YEAST Cystathionine gamma-synthase OS=Saccharomyces cerevisiae (strain ATCC 204508 / S288c) OX=559292 GN=STR2 PE=1 SV=1	3.8	72.35	7.4065	394740	0
sp|P22133|MDHC_YEAST	1	sp|P22133|MDHC_YEAST Malate dehydrogenase, cytoplasmic OS=Saccharomyces cerevisiae (strain ATCC 204508 / S288c) OX=559292 GN=MDH2 PE=1 SV=2	4.8	40.73	7.3599	2129100	0
sp|P39105|PLB1_YEAST	1	sp|P39105|PLB1_YEAST Lysophospholipase 1 OS=Saccharomyces cerevisiae (strain ATCC 204508 / S288c) OX=559292 GN=PLB1 PE=1 SV=2	4.5	71.667	7.3171	680540	0
sp|P54784|ORC1_YEAST	1	sp|P54784|ORC1_YEAST Origin recognition complex subunit 1 OS=Saccharomyces cerevisiae (strain ATCC 204508 / S288c) OX=559292 GN=ORC1 PE=1 SV=1	1.1	104.4	6.5318	2291000	2
sp|P38853|KEL1_YEAST	1	sp|P38853|KEL1_YEAST Kelch repeat-containing protein 1 OS=Saccharomyces cerevisiae (strain ATCC 204508 / S288c) OX=559292 GN=KEL1 PE=1 SV=1	0.9	131.09	6.4293	49601000	1
sp|P13185|KIN1_YEAST	1	sp|P13185|KIN1_YEAST Serine/threonine protein kinase KIN1 OS=Saccharomyces cerevisiae (strain ATCC 204508 / S288c) OX=559292 GN=KIN1 PE=1 SV=3	2.9	120.07	6.3575	1255000	0
sp|Q05164|HPF1_YEAST	1	sp|Q05164|HPF1_YEAST Haze protective factor 1 OS=Saccharomyces cerevisiae (strain ATCC 204508 / S288c) OX=559292 GN=HPF1 PE=1 SV=2	3.2	94.704	6.3575	4164500	0

sp|P05986|KAPC_YEAST	1	sp|P05986|KAPC_YEAST cAMP-dependent protein kinase type 3 OS=Saccharomyces cerevisiae (strain ATCC 204508 / S288c) OX=559292 GN=TPK3 PE=1 SV=2	3.3	45.977	6.2288	0	1
sp|Q03002|FRK1_YEAST	1	sp|Q03002|FRK1_YEAST Fatty acyl-CoA synthetase and RNA processing-associated kinase 1 OS=Saccharomyces cerevisiae (strain ATCC 204508 / S288c) OX=559292 GN=FRK1 PE=1 SV=1	2.8	97.692	6.1776	520910000	2
sp|P32569|MED17_YEAST	1	sp|P32569|MED17_YEAST Mediator of RNA polymerase II transcription subunit 17 OS=Saccharomyces cerevisiae (strain ATCC 204508 / S288c) OX=559292 GN=SRB4 PE=1 SV=1	3.5	78.475	6.1479	2786200	1
sp|P36169|SKG1_YEAST	1	sp|P36169|SKG1_YEAST Suppressor of lethality of KEX2 GAS1 double null mutant protein 1 OS=Saccharomyces cerevisiae (strain ATCC 204508 / S288c) OX=559292 GN=SKG1 PE=1 SV=2	3.1	39.829	6.0796	1232700	0
sp|P53074|YGY0_YEAST	1	sp|P53074|YGY0_YEAST Uncharacterized protein YGL230C OS=Saccharomyces cerevisiae (strain ATCC 204508 / S288c) OX=559292 GN=YGL230C PE=4 SV=1	15	17.262	6.053	1005300	1
sp|P53966|KTR5_YEAST	1	sp|P53966|KTR5_YEAST Probable mannosyltransferase KTR5 OS=Saccharomyces cerevisiae (strain ATCC 204508 / S288c) OX=559292 GN=KTR5 PE=1 SV=1	4.2	61.727	6.053	601020	1
sp|Q05473|MRX8_YEAST	1	sp|Q05473|MRX8_YEAST MIOREX complex component 8 OS=Saccharomyces cerevisiae (strain ATCC 204508 / S288c) OX=559292 GN=MRX8 PE=1 SV=1	7	35.614	6.044	165700000	1
sp|Q06698|YL419_YEAST	1	sp|Q06698|YL419_YEAST Putative ATP-dependent RNA helicase YLR419W OS=Saccharomyces cerevisiae (strain ATCC 204508 / S288c) OX=559292 GN=YLR419W PE=1 SV=1	0.7	163.04	5.9965	3073800	1
sp|P40013|BIM1_YEAST	1	sp|P40013|BIM1_YEAST Protein BIM1 OS=Saccharomyces cerevisiae (strain ATCC 204508 / S288c) OX=559292 GN=BIM1 PE=1 SV=1	3.2	38.361	5.9814	303360	0
sp|P53289|RTS3_YEAST	1	sp|P53289|RTS3_YEAST Protein phosphatase type 2A regulatory subunit RTS3 OS=Saccharomyces cerevisiae (strain ATCC 204508 / S288c) OX=559292 GN=RTS3 PE=1 SV=1	4.2	29.263	5.9814	575230	0
sp|Q06632|CFT1_YEAST	1	sp|Q06632|CFT1_YEAST Protein CFT1 OS=Saccharomyces cerevisiae (strain ATCC 204508 / S288c) OX=559292 GN=CFT1 PE=1 SV=1	1.7	153.4	5.9483	28370000	1
sp|Q07555|YD129_YEAST	1	sp|Q07555|YD129_YEAST Uncharacterized protein YDL129W OS=Saccharomyces cerevisiae (strain ATCC 204508 / S288c) OX=559292 GN=YDL129W PE=1 SV=1	6.5	32.916	5.9306	15652000	1
sp|P38970|HAL5_YEAST	1	sp|P38970|HAL5_YEAST Serine/threonine-protein kinase HAL5 OS=Saccharomyces cerevisiae (strain ATCC 204508 / S288c) OX=559292 GN=HAL5 PE=1 SV=2	1.3	95.454	5.9093	126550000	0
sp|O14467|MBF1_YEAST	1	sp|O14467|MBF1_YEAST Multiprotein-bridging factor 1 OS=Saccharomyces cerevisiae (strain ATCC 204508 / S288c) OX=559292 GN=MBF1 PE=1 SV=2	6.6	16.403	5.9076	4276900	1
sp|Q12050|ELG1_YEAST	1	sp|Q12050|ELG1_YEAST Telomere length regulation protein ELG1 OS=Saccharomyces cerevisiae (strain ATCC 204508 / S288c) OX=559292 GN=ELG1 PE=1 SV=1	2	91.26	5.8896	41766000	2
sp|Q3E7B3|YPR08_YEAST	1	sp|Q3E7B3|YPR08_YEAST Uncharacterized protein YPR108W-A OS=Saccharomyces cerevisiae (strain ATCC 204508 / S288c) OX=559292 GN=YPR108W-A PE=4 SV=1	17.1	7.7562	5.8728	7126400	1
sp|Q02457|TBF1_YEAST	1	sp|Q02457|TBF1_YEAST Protein TBF1 OS=Saccharomyces cerevisiae (strain ATCC 204508 / S288c) OX=559292 GN=TBF1 PE=1 SV=2	7.8	62.823	5.8357	1209600	2
sp|Q02796|LGE1_YEAST	1	sp|Q02796|LGE1_YEAST Transcriptional regulatory protein LGE1 OS=Saccharomyces cerevisiae (strain ATCC 204508 / S288c) OX=559292 GN=LGE1 PE=1 SV=1	6	37.309	5.8285	820160	0
sp|Q04399|GMC1_YEAST	1	sp|Q04399|GMC1_YEAST Putative multicopper oxidase GMC1 OS=Saccharomyces cerevisiae (strain ATCC 204508 / S288c) OX=559292 GN=GMC1 PE=1 SV=1	3.3	69.28	5.8285	7967600	0
sp|P40456|ESL1_YEAST	1	sp|P40456|ESL1_YEAST EST/SMG-like protein 1 OS=Saccharomyces cerevisiae (strain ATCC 204508 / S288c) OX=559292 GN=ESL1 PE=1 SV=1	0.9	128.73	5.8117	692600	0
sp|P38705|SYSM_YEAST	1	sp|P38705|SYSM_YEAST Serine--tRNA ligase, mitochondrial OS=Saccharomyces cerevisiae (strain ATCC 204508 / S288c) OX=559292 GN=DIA4 PE=1 SV=1	2.9	50.389	5.8078	2341600	1

sp|P53253|NNF2_YEAST	1	sp|P53253|NNF2_YEAST Protein NNF2 OS=Saccharomyces cerevisiae (strain ATCC 204508 / S288c) OX=559292 GN=NNF2 PE=1 SV=1	1.3	106.46	5.8024	430550	0
sp|P40343|VPS27_YEAST	1	sp|P40343|VPS27_YEAST Vacuolar protein sorting-associated protein 27 OS=Saccharomyces cerevisiae (strain ATCC 204508 / S288c) OX=559292 GN=VPS27 PE=1 SV=3	1.8	70.973	5.785	1838300	1
sp|Q04500|UTP14_YEAST	1	sp|Q04500|UTP14_YEAST U3 small nucleolar RNA-associated protein 14 OS=Saccharomyces cerevisiae (strain ATCC 204508 / S288c) OX=559292 GN=UTP14 PE=1 SV=1	2.7	103.02	5.7841	385490	1
sp|P28241|IDH2_YEAST	1	sp|P28241|IDH2_YEAST Isocitrate dehydrogenase [NAD] subunit 2, mitochondrial OS=Saccharomyces cerevisiae (strain ATCC 204508 / S288c) OX=559292 GN=IDH2 PE=1 SV=1	4.9	39.739	5.7799	3045800	0
sp|P50087|MIC26_YEAST	1	sp|P50087|MIC26_YEAST MICOS subunit MIC26 OS=Saccharomyces cerevisiae (strain ATCC 204508 / S288c) OX=559292 GN=MIC26 PE=1 SV=1	7.7	26.912	5.7799	582660	0
sp|Q03661|ESC1_YEAST	1	sp|Q03661|ESC1_YEAST Silent chromatin protein ESC1 OS=Saccharomyces cerevisiae (strain ATCC 204508 / S288c) OX=559292 GN=ESC1 PE=1 SV=1	1.1	187.14	5.7799	788120	0
sp|Q12125|GET4_YEAST	1	sp|Q12125|GET4_YEAST Golgi to ER traffic protein 4 OS=Saccharomyces cerevisiae (strain ATCC 204508 / S288c) OX=559292 GN=GET4 PE=1 SV=1	5.8	36.28	5.7799	15684000	0
sp|P18634|ART10_YEAST	1	sp|P18634|ART10_YEAST Arrestin-related trafficking adapter 10 OS=Saccharomyces cerevisiae (strain ATCC 204508 / S288c) OX=559292 GN=ART10 PE=1 SV=3	2.5	59.757	5.7798	1907700	0
sp|P0CE00|MPH3_YEAST	1	sp|P0CE00|MPH3_YEAST Alpha-glucosides permease MPH3 OS=Saccharomyces cerevisiae (strain ATCC 204508 / S288c) OX=559292 GN=MPH3 PE=1 SV=1	1.3	67.256	5.7718	754450	1
sp|P38144|ISW1_YEAST	1	sp|P38144|ISW1_YEAST ISWI chromatin-remodeling complex ATPase ISW1 OS=Saccharomyces cerevisiae (strain ATCC 204508 / S288c) OX=559292 GN=ISW1 PE=1 SV=2	2.1	131.1	5.7716	18000000000	5
sp|P13574|STE12_YEAST	1	sp|P13574|STE12_YEAST Protein STE12 OS=Saccharomyces cerevisiae (strain ATCC 204508 / S288c) OX=559292 GN=STE12 PE=1 SV=1	3.5	77.866	5.7603	427490000	1
sp|Q03714|USA1_YEAST	1	sp|Q03714|USA1_YEAST U1 SNP1-associating protein 1 OS=Saccharomyces cerevisiae (strain ATCC 204508 / S288c) OX=559292 GN=USA1 PE=1 SV=1	2.9	96.652	5.7579	676830	1
sp|P16547|OM45_YEAST	1	sp|P16547|OM45_YEAST Mitochondrial outer membrane protein OM45 OS=Saccharomyces cerevisiae (strain ATCC 204508 / S288c) OX=559292 GN=OM45 PE=1 SV=2	6.1	44.58	5.7362	432910	1
sp|P53971|FAP1_YEAST	1	sp|P53971|FAP1_YEAST FKBP12-associated protein 1 OS=Saccharomyces cerevisiae (strain ATCC 204508 / S288c) OX=559292 GN=FAP1 PE=1 SV=1	2.5	108.49	5.7362	2253200	2
sp|P25037|UBP1_YEAST	1	sp|P25037|UBP1_YEAST Ubiquitin carboxyl-terminal hydrolase 1 OS=Saccharomyces cerevisiae (strain ATCC 204508 / S288c) OX=559292 GN=UBP1 PE=1 SV=2	2.8	92.768	5.7302	7906500	0

TABLE S2 Strains used in this study.

Strain	Genotype	Source
S. cerevisiae		
AN120	*MATα/MATa ARG4/arg4-NspI his3ΔSK/his3ΔSK ho::LYS2/ho::LYS2 leu2/leu2 lys2/lys2 RME1/rme1::LEU2 trp1::hisG/trp1::hisG ura3/ura3*	38
AN117-16D	*MATa ura3 leu2 trp1 his3ΔSK lys2 ho::LYS2*	38
C.glabrata		
KUE100	*his3 yku80::SAT1 flipper*	39
E. coli		
BL21(DE3)	F^{-} *ompT* $hsdS_{B}$ (r_{B}^{-} m_{B}^{-}) *gal dcm* λ(DE3)	Invitrogen

TABLE S3 Oligo nucleotides used in this study.

Name	Sequence (5’-3’)	Restriction enzyme site
R1	CGCGGGATCCATGGCTAGAACTTTCTTTGT	*Bam*HI
R2	CGCGGAGCTCTTAGTTTCTAGAGTTGATGA	*Sac*I
R3	CGTTGTCGCTTACGCACCAGTCTGGGCCA	-
R4	TGGCCCAGACTGGTGCGTAAGCGACAACG	-
R5	GCGCGAGCTCTACCAATGTTCCTAACGGGA	*Sac*I
R6	GCGCACTAGTTTTTAGTTTATGTATGTGTT	*Spe*I
R7	CGCGATCGATATGGCTAGAACTTTCTTTGT	*Cla*I
R8	CGCGCTCGAGGTTTCTAGAGTTGATGATAT	*Xho*I
R9	GCGCGGATCCATGGCTAAGCTAAAAGAACC	*Bam*HI
R10	GCGCAAGCTTCTCTTTTATGATCCCACTTA	*Hin*dIII
clb2-F2	GGGCTTTAAAGGTTAGAAAAAACGGCTATGATATAATGACCTTGCATGAACGGATCCCCGGGTTAATTAA	-
clb2-R1	GGACATTTATCGATTATCGTTTTAGATATTTTAAGCATCTGCCCCTCTTCGAATTCGAGCTCGTTTAAAC	-
tpi1-up-F	AAGGATGAGCCAAGAATAAGGGAACAAGATTTTGGTAATT	-
tpi1-up-R	TTATATAATTATATTAATCTTTTAGTTTATGTATGTGTTT	-
tpi1-down-F	GATTAATATAATTATATAAAAATATTATCTTCTTTTCTTT	-
tpi1-down-R	CTCAAATATTAAATGCGATCCAACGATCAAGAACAGACTA	-
VF	TGAAGGGAAGCCTGGAAAATGTTATCTGCAATATCGGGTT	-
VR	CATCGCAGCAAGGGATGAAACAGATGGCACTGATTTTGCT	-
gRNA-F	GCAGTGAAAGATAAATGATCGAATTGAGAATCTTATACGGGTTTTAGAGCTAGAAATAGC	-
gRNA-R	GCTATTTCTAGCTCTAAAACCCGTATAAGATTCTCAATTCGATCATTTATCTTTCACTGC	-

TABLE S4 Plasmids used in this study.

Name	Parental plasmid	Description	Cloning sites
pET28a-TPI1	pET28a	Gene, *TPI1*; Primers, R1 and R2	*Bam*HI/*Sac*I
pET28a-$TPI1^{E165A}$	pET28a-TPI1	pET28a-TPI1 was mutated with R3 and R4	-
pRS316-TPI1pr-FLAG	pRS426-TPI1pr-FLAG * and pRS316 [41]	Gene, *TPI1* promoter and FLAG derived from pRS426-TPI1pr-FLAG	*Sac*I/KpnI
pRS316-TPI1pr-TPI1-FLAG	pRS316-TPI1pr-FLAG	Gene, *TPI1*; Primers, R7 and R8	*Cla*I/*Xho*I
pRS316-TPI1pr-PwTPIA-FLAG	pRS316-TPI1pr-FLAG	Gene, *PwTPIA*; Primers, R9 and R10	*Bam*HI/*Hin*dIII
p426-SNR52p-gRNA-TPI1	p426-SNR52p-gRNA	Gene, *TPI1* targeting gRNA; Primers, gRNA-F and gRNA-R	-

* Refer Methods
